# Quantitative classification and radiomics of [^18^F]FDG-PET/CT in indeterminate thyroid nodules

**DOI:** 10.1007/s00259-022-05712-0

**Published:** 2022-02-09

**Authors:** Elizabeth J. de Koster, Wyanne A. Noortman, Jacob M. Mostert, Jan Booij, Catherine B. Brouwer, Bart de Keizer, John M. H. de Klerk, Wim J. G. Oyen, Floris H. P. van Velden, Lioe-Fee de Geus-Oei, Dennis Vriens, for the EfFECTS trial study group

**Affiliations:** 1 Department of Radiology and Nuclear Medicine, Radboud University Medical Center, Nijmegen, the Netherlands; 2 Department of Radiology, Section of Nuclear Medicine, Leiden University Medical Center, Leiden, the Netherlands; 3 Biomedical Photonic Imaging Group, University of Twente, Enschede, the Netherlands; 4 Delft University of Technology, Delft, the Netherlands; 5 Department of Radiology and Nuclear Medicine, Amsterdam University Medical Centers, Location Academic Medical Center, Amsterdam, the Netherlands; 6 Department of Internal Medicine, OLVG Hospital, Amsterdam, the Netherlands; 7 Department of Radiology and Nuclear Medicine, University Medical Centre Utrecht, Utrecht, the Netherlands; 8 Department of Nuclear Medicine, Meander Medical Centre, Amersfoort, the Netherlands; 9 Department of Radiology and Nuclear Medicine, Rijnstate Hospital, Arnhem, the Netherlands; 10 Department of Biomedical Sciences and Humanitas Clinical and Research Centre, Department of Nuclear Medicine, Humanitas University, Milan, Italy

**Keywords:** [^18^F]FDG-PET/CT, Indeterminate, Thyroid nodule, Thyroid carcinoma, Thyroid cytology, Quantitative, Standardised uptake value, Radiomics

## Abstract

**Purpose:**

To evaluate whether quantitative [^18^F]FDG-PET/CT assessment, including radiomic analysis of [^18^F]FDG-positive thyroid nodules, improved the preoperative differentiation of indeterminate thyroid nodules of non-Hürthle cell and Hürthle cell cytology.

**Methods:**

Prospectively included patients with a Bethesda III or IV thyroid nodule underwent [^18^F]FDG-PET/CT imaging. Receiver operating characteristic (ROC) curve analysis was performed for standardised uptake values (SUV) and SUV-ratios, including assessment of SUV cut-offs at which a malignant/borderline neoplasm was reliably ruled out (≥ 95% sensitivity). [^18^F]FDG-positive scans were included in radiomic analysis. After segmentation at 50% of SUV_peak_, 107 radiomic features were extracted from [^18^F]FDG-PET and low-dose CT images. Elastic net regression classifiers were trained in a 20-times repeated random split. Dimensionality reduction was incorporated into the splits. Predictive performance of radiomics was presented as mean area under the ROC curve (AUC) across the test sets.

**Results:**

Of 123 included patients, 84 (68%) index nodules were visually [^18^F]FDG-positive. The malignant/borderline rate was 27% (33/123). SUV-metrices showed AUCs ranging from 0.705 (95% CI, 0.601–0.810) to 0.729 (0.633–0.824), 0.708 (0.580–0.835) to 0.757 (0.650–0.864), and 0.533 (0.320–0.747) to 0.700 (0.502–0.898) in all (*n* = 123), non-Hürthle (*n* = 94), and Hürthle cell (*n* = 29) nodules, respectively. At SUV_max_, SUV_peak_, SUV_max_-ratio, and SUV_peak_-ratio cut-offs of 2.1 g/mL, 1.6 g/mL, 1.2, and 0.9, respectively, sensitivity of [^18^F]FDG-PET/CT was 95.8% (95% CI, 78.9–99.9%) in non-Hürthle cell nodules. In Hürthle cell nodules, cut-offs of 5.2 g/mL, 4.7 g/mL, 3.4, and 2.8, respectively, resulted in 100% sensitivity (95% CI, 66.4–100%). Radiomic analysis of 84 (68%) [^18^F]FDG-positive nodules showed a mean test set AUC of 0.445 (95% CI, 0.290–0.600) for the PET model.

**Conclusion:**

Quantitative [^18^F]FDG-PET/CT assessment ruled out malignancy in indeterminate thyroid nodules. Distinctive, higher SUV cut-offs should be applied in Hürthle cell nodules to optimize rule-out ability. Radiomic analysis did not contribute to the additional differentiation of [^18^F]FDG-positive nodules.

**Trial registration number:**

This trial is registered with ClinicalTrials.gov: NCT02208544 (5 August 2014), https://clinicaltrials.gov/ct2/show/NCT02208544.

**Supplementary Information:**

The online version contains supplementary material available at 10.1007/s00259-022-05712-0.

## Introduction

An accurate diagnostic workup of cytologically indeterminate thyroid nodules is crucial to prevent futile diagnostic surgeries for benign nodules as well as to ensure timely diagnosis of malignant or borderline tumours. Including cytology with atypia of undetermined significance or follicular lesions of undetermined significance (Bethesda III, AUS/FLUS) and cytology suspicious for a follicular neoplasm (Bethesda IV, FN/SFN) or Hürthle cell neoplasm (Bethesda IV, HCN/SHCN), indeterminate thyroid nodules have an approximate 25% risk of malignancy [1–3]. Our recent randomised controlled trial showed that *visual* assessment of [^18^F]-2-fluoro-2-deoxy-D-glucose positron emission tomography/computed tomography ([^18^F]FDG-PET/CT) reliably rules out thyroid malignancy in [^18^F]FDG-negative indeterminate nodules, with a reported 94% sensitivity and 31% benign call rate (i.e., fraction of negative tests). As such, [^18^F]FDG-PET/CT-driven management resulted in an oncologically safe 40% reduction in futile diagnostic surgeries for benign nodules. With a limited 40% specificity, visual [^18^F]FDG-PET/CT assessment increased the post-test risk of malignancy but appeared unable to fully risk-stratify the approximately two-thirds visually [^18^F]FDG-*positive* nodules [4]. These results validated the findings from previously published, smaller, non-randomised studies [5–12].

Part of this limited specificity is explained by the proportion of HCN/SHCN cytology, which varied from 21 to 52% in previous PET/CT studies including 23% in our trial [4–6]. Hürthle cell neoplasms, defined as tumours composed of > 75% Hürthle cells, constitute an extraordinary subgroup: following the abundance of mitochondria in their oxyphilic follicular-derived cells, nearly all of these neoplasms are strongly [^18^F]FDG-positive [4, 13–15]. As such, visual [^18^F]FDG-PET/CT assessment cannot differentiate between benign and malignant Hürthle cell nodules. We previously advocated that a *visual* [^18^F]FDG-PET/CT-driven diagnostic workup should be limited to non-Hürthle cell Bethesda III/IV nodules to optimize therapeutic yield [4].

The risk of malignancy in nodules with HCN/SHCN cytology appears lower than in FN/SFN cytology, but Hürthle cell carcinomas typically show more aggressive behaviour and less favourable prognosis than their non-oncocytic follicular counterparts [13, 15]. This underlines the currently unmet need for an accurate diagnostic workup for this subgroup.

Several studies have reported the *quantitative* assessment of [^18^F]FDG-PET/CT images using the standardised uptake value (SUV, g/mL) of the indeterminate thyroid nodule, most frequently reported as the maximum SUV (SUV_max_) [8, 10–12, 16]. A higher SUV_max_ was generally reported in thyroid malignancies than in benign lesions [5–7, 9]. Threshold analysis using a SUV_max_ cut-off of 5 g/mL resulted in 42–80% sensitivity and 41–91% specificity to detect malignancy [6, 8, 10]. To the best of our knowledge, there is no evidence on the quantitative assessment of [^18^F]FDG-PET/CT in Hürthle cell nodules.

In addition to the traditional quantitative PET features such as the SUV_max_, PET/CT images harbour an abundance of information inside the myriad of voxels that could be identified using radiomics [17]. In radiomics, large amounts of quantitative features are extracted from medical images, aiming to find stable and clinically relevant image-derived biomarkers that may provide new insights in tumour biology and guide patient management [18]. After a number of studies suggested that radiomic analysis could contribute to the differentiation of [^18^F]FDG-positive thyroid incidentalomas, one study recently also indicated its potential in the diagnosis of indeterminate nodules [19–24].

In the current study, we sought to optimize the [^18^F]FDG-PET/CT-driven differentiation of indeterminate thyroid nodules through quantitative [^18^F]FDG-PET/CT assessment including radiomic analysis, with particular attention for the separate assessment of non-Hürthle and Hürthle cell nodules. We aimed to rule out malignancy and decrease the false-positive rate as compared to visual [^18^F]FDG-PET/CT assessment. We ultimately aimed to further prevent futile surgeries for benign, [^18^F]FDG-positive indeterminate nodules.

## Methods

### Study design and patient selection

**Fig. 1:**
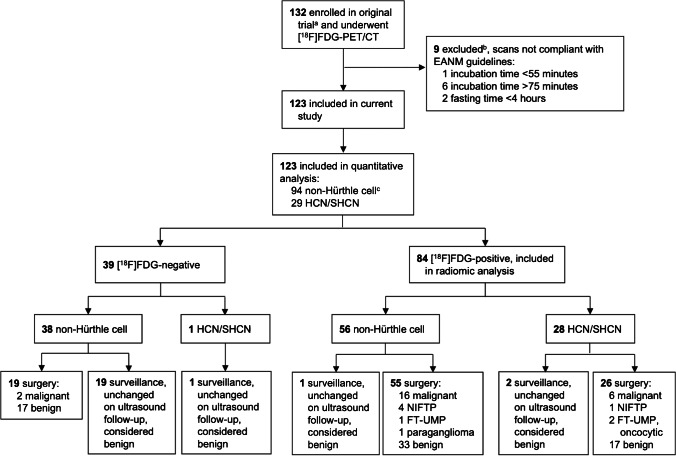
Study flowchart. ^a^Patient screening for the original trial, including eligibility criteria, were previously published [4]. ^b^Baseline characteristics of included patients (Table 1) were similar to those of excluded patients (Supplementary table 3). ^c^non-Hürthle cell nodules comprise nodules of AUS/FLUS (*n* = 55) and FN/SFN (*n* = 39) cytology. AUS/FLUS, atypia of undetermined significance or follicular lesion of undetermined significance. FN/SFN, cytology (suspicious for a) follicular neoplasm. FT-UMP, follicular tumour of uncertain malignant potential. HCN/SHCN, (suspicious for a) Hürthle cell neoplasm. NIFTP, non-invasive follicular thyroid neoplasm with papillary-like nuclear features

All patients who participated in a randomised controlled multicentre trial (ClinicalTrials.gov NCT02208544) on the efficacy of [^18^F]FDG-PET/CT in cytologically indeterminate thyroid nodules (*EfFECTS*) were assessed for eligibility for the current study. The *EfFECTS* trial was conducted in eight academic and seven community hospitals in the Netherlands with a high level of experience in the diagnosis and treatment of thyroid nodules and differentiated thyroid carcinoma (Supplementary data). [^18^F]FDG-PET/CT was performed in 132 patients with a solitary nodule or dominant nodule in multinodular disease from which indeterminate cytology was obtained, defined as at least two Bethesda III or one Bethesda IV cytology result (confirmed on central review). Based on cytology, clinical characteristics, and ultrasound features, diagnostic thyroid surgery was scheduled in all patients, in accordance with current international guidelines [25]. Further inclusion and exclusion criteria of the *EfFECTS* trial, and its comprehensive study procedures were previously reported [4]. Patients from the original study were only eligible for inclusion in the *current* study if their [^18^F]FDG-PET/CT scan was acquired with strict adherence to the EANM guidelines, including a patient fasting time of at least 4 h and an acquisition time between 55 and 75 min (Fig. 1) [26]. Written informed consent was obtained from all participants prior to any study activity. The trial was approved by the Medical Research Ethics Committee on Research Involving Human Subjects region Arnhem-Nijmegen, Nijmegen, the Netherlands. The funder of the trial had no influence on the design or conduct of the trial and was not involved in the collection or analysis of the data, or in the writing of the manuscript.

### Image acquisition and reconstruction

During the *EfFECTS* trial, all participants underwent an [^18^F]FDG-PET/CT covering skull-base to upper thorax. These scans were acquired by 20 different scanners at 12 EARL-accredited study sites (Supplementary table 1) using a standard acquisition and reconstruction protocol in accordance with European Association of Nuclear Medicine (EANM) guidelines [26]. Patients were advised to fast for at least 6 h. Serum glucose levels were between 4 and 11 mmol/L. PET-acquisition was scheduled 60 (55–75) minutes after intravenous bolus administration of [^18^F]FDG. The administered activity was dependent on body weight, scan speed, bed overlap, and scanner sensitivity, equivalent to 3.45 MBq/kg (4 min/bed, < 25% bed overlap). Low-dose, non-contrast-enhanced CT (ldCT) scans were acquired for attenuation correction of PET images. Additional details on patient preparation, data acquisition, image reconstruction, and image processing are reported in Supplementary table 2.

### [^18^F]FDG-PET/CT quantitative analysis

**Fig. 2:**
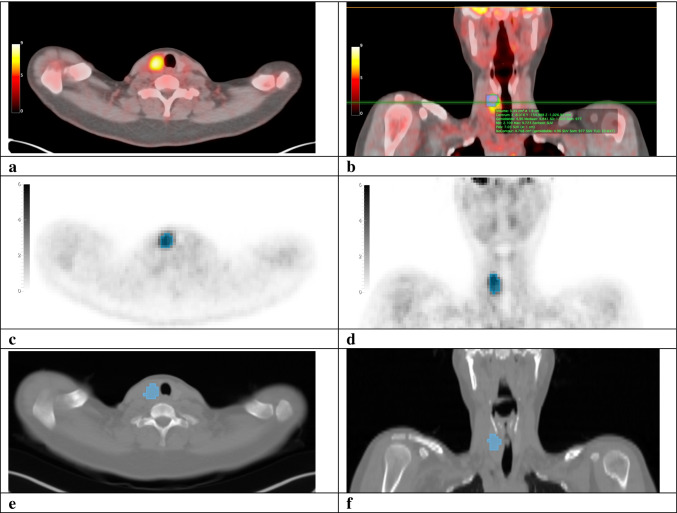
Quantitative [^18^F]FDG-PET/CT assessment and delineation of the VOI for radiomic analysis. Transverse and coronal [^18^F]FDG-PET/CT (**a**, **b**), maximum intensity projection (MIP) (**c**, **d**) and low-dose CT (**e**, **f**) images of a patient with a solitary, 30 mm Bethesda III thyroid nodule in the right lobe. Visual assessment (**a**) of the [^18^F]FDG-PET/CT showed an [^18^F]FDG-positive index nodule. Quantitative assessment (**b**) demonstrated a SUV_max_ of 9.7 g/mL and SUV_peak_ of 7.0 g/mL of the index nodule, and a SUV_max_ of 1.6 g/mL in the background of surrounding normal thyroid tissue. Consequently, the SUV_max_-ratio and SUV_peak_-ratio were 6.1 (9.7/1.6) and 4.4 (7.0/1.6), respectively. For radiomic analysis, VOIs were delineated on the [^18^F]FDG-PET scans using an isocontour that applies a threshold of 50% of the SUV_peak_, corrected for local background (**c**, **d**) [29]. Boxing was applied to exclude [^18^F]FDG-positive tissue surrounding the index nodule and ldCT images were used as a visual reference (**e**, **f**). VOIs delineated on the PET images were resampled with a nearest neighbour algorithm to derive the ldCT VOIs

Quantitative image analyses were performed using OsiriX Lite DICOM-viewer (Pixmeo SARL, Bernex, Switzerland). SUV-computation was validated after each mandatory software version update. All scans were centrally assessed by two independent, experienced nuclear medicine physicians (DV, LF). They were blinded to patient allocation and all clinical and cytological data except for the ultrasonographic size and location of the index nodule, to ensure its correct identification. For the visual assessment, any focal [^18^F]FDG-uptake within the thyroid that was visually higher than the physiological background [^18^F]FDG-uptake of the surrounding normal thyroid tissue and that corresponded to the index nodule in size and location, was considered positive. The SUV_max_ and peak SUV (SUV_peak_, defined as the maximum average SUV within a 1 cm^3^ spherical volume) of the index nodule were semi-automatically measured (Fig. 2) [27]. Body weight corrected values were used. The SUV_max_-ratio and SUV_peak_-ratio were respectively calculated by dividing the SUV_max_ and SUV_peak_ of the nodule by the background SUV_max_ of normal thyroid tissue in the contralateral lobe. [^18^F]FDG-positive foci in the thyroid that did not correspond to the index nodule in size and location (i.e., thyroid incidentalomas) were not analysed.

### Radiomic analysis

All visually [^18^F]FDG-positive nodules, defined as index nodules with focal [^18^F]FDG-uptake that was visually higher than the background [^18^F]FDG-uptake in the surrounding normal thyroid tissue, were included in the radiomic analysis.

#### Volume of interest definition

Volumes of interest (VOI) were delineated semi-automatically around visually [^18^F]FDG-positive nodules using 3DSlicer (version 4.11; slicer.org) and in-house built software implemented in Python (version 3.6.10; Python Software Foundation, Wilmington, Delaware) [28]. VOIs were delineated on the [^18^F]FDG-PET scans using an isocontour that applies a threshold of 50% of the SUV_peak_, corrected for local background activity (Fig. 2) [29]. Boxing was applied to exclude [^18^F]FDG-positive tissue surrounding the index nodule and ldCT images were used as a visual reference. VOIs delineated on the PET images were resampled with a nearest neighbour algorithm to derive the ldCT VOIs. Potential mismatch was evaluated visually: no corrections were required.

#### Image processing and radiomic feature extraction

Radiomic features were extracted from the VOIs on both the interpolated PET (4 × 4 × 4 mm^3^) and the ldCT (2 × 2 × 2 mm^3^) images using PyRadiomics (version 2.1.2 in Python version 3.6.10; pyradiomics.readthedocs.io) [30]. From both PET and CT images, 107 standardised features were extracted: 14 shape features, 18 intensity features, and 75 texture features (24 grey level co-occurrence matrix (GLCM), 16 grey level run length matrix, 16 grey level size zone matrix, 14 grey level dependence matrix, five neighbouring grey tone difference matrix). For PET, the total lesion glycolysis (TLG, defined as the product of the SUV_mean_ and the metabolic tumour volume) was also added. A fixed bin size of 0.5 g/mL for PET and 25 HU for ldCT was used (Supplementary table 2) [31].

### Reference standard

During participation in the *EfFECTS* trial, patients were advised to refrain from the scheduled diagnostic surgery when they were allocated to the [^18^F]FDG-PET/CT-driven group and the index nodule was visually [^18^F]FDG-negative. These patients remained under active surveillance and had at least a follow-up ultrasound examination after 12 months. All other patients were advised to proceed to the scheduled diagnostic surgery and were treated according to current guidelines [4, 32]. This resulted in the following reference standard for the current study: benign nodules were defined either as benign on final histopathology (i.e., hyperplastic nodules, follicular adenoma or Hürthle cell adenoma) or as index nodules that remained unchanged in size and appearance on ultrasound follow-up, in accordance with definitions from the *EfFECTS* trial. Malignancies and borderline nodules were defined as index nodules that were histopathologically diagnosed as thyroid carcinoma or borderline tumours, the latter including non-invasive follicular thyroid neoplasm with papillary-like nuclear features (NIFTP), follicular tumour of uncertain malignant potential (FT-UMP), and paraganglioma. Throughout the manuscript, malignant and borderline lesions are grouped, as diagnostic surgery is considered the right course of treatment for all these lesions according to current insights. Incidentally detected (micro)carcinomas or borderline tumours located outside the index nodule were not considered for the reference standard. Blinded central revision of all cyto- and histopathology was performed by a dedicated thyroid pathologist. In case of discordance with the local histopathologist, a third pathologist was consulted and consensus was reached.

### Outcomes

The primary outcome of the study was the diagnostic accuracy of quantitative [^18^F]FDG-PET/CT assessment and radiomics in non-Hürthle cell (defined as AUS/FLUS and FN/SFN cytology) and Hürthle cell (defined as HCN/SHCN cytology) nodules. True-positive and false-negative were respectively defined as test-positive and test-negative histopathologically malignant/borderline nodules. False-positive and true-negative were respectively defined as test-positive and test-negative benign nodules.

### Statistical and radiomic analysis

Categorical data were expressed as absolute and relative (%) frequencies, and compared using Pearson’s chi-squared or Fisher’s exact tests, where appropriate. Continuous data were assessed for log-normality, expressed using mean ± standard deviation or median (interquartile range), and compared using independent samples *t*-tests or Mann–Whitney *U* tests when (log-)normally or non-normally distributed, respectively. Receiver operator characteristic (ROC) curve analysis was performed for the SUV_max_, SUV_peak_, SUV_max_-ratio, and SUV_peak_-ratio, using the area under the curve (AUC) to describe the overall diagnostic accuracy. Next, for each of the SUV-metrices, the cut-off value was determined at which an optimal test sensitivity was found, defined as a sensitivity ≥ 95%. This is in accordance with the current ATA recommendations that a useful rule-out test is characterised by a negative predictive value (NPV) similar to a Bethesda II cytological diagnosis (i.e., 96%) [25]. At these SUV cut-offs, we assessed the benign call rate, representing the rate of potentially avoidable diagnostic surgeries. Sensitivity, specificity, negative and positive predictive value (PPV), benign call rate, and 95% confidence intervals (CI) were calculated using the traditional formulas and β-distribution (Clopper-Pearson interval), respectively. Subgroup analysis was performed for [^18^F]FDG-positive non-Hürthle cell nodules. Data collection was performed using Castor EDC (Castor EDC, Amsterdam, the Netherlands). Statistical analysis was performed in SPSS Statistics (version 26; IBM Corp, Armonk, NY, USA).

#### Radiomic classifier

Radiomic analysis was performed in Python (version 3.6.10) and R (version 3.6.0; R Foundation for Statistical Computing, Vienna, Austria). In Python, an elastic net regression classifier was trained and evaluated in a 20-times repeated random split, in which the dataset was split in 80% training and 20% test data. Since the number of extracted features exceeded the number of patients in the dataset, dimensionality reduction incorporating redundancy filtering and factor analysis of radiomic features was performed for each split on the training set using FMradio (Factor Modelling for Radiomics Data) R-package (version 1.1.1) [33]. One factor was selected for every ten subjects in the training set (Details provided in Supplementary table 2) [18]. Factors for the training and test set were calculated. The factors of the training set were used as input for the elastic net regression classifier. The predictive performance of the model is expressed as the mean AUC of the ROC curve over the 20 splits for the test sets. The 95% CIs were constructed using a corrected resampled *t*-test [34]. Classification models were trained on PET features and subsequently on PET and ldCT features. The definitions of the factors in the model were determined based on the underlying clusters of features in the different folds. Subgroup analysis was performed for nodules meeting the minimal size recommendation for radiomic analysis of 64 voxels per VOI [35]. The TRIPOD statement (transparent reporting of multivariable prediction model for individual prognosis or diagnosis, version 1 October 2020) was used (IBSI reporting guidelines, Supplementary table 2) [36].

## Results

### Patients

The current study included 123 patients between 1 July 2015 and 16 October 2018 (Fig. 1, Table 1). Cytology was AUS/FLUS in 55 (45%), FN/SFN in 39 (32%), and HCN/SHCN in 29 (24%) patients. One hundred (81%) patients underwent diagnostic surgery and 23 (19%) underwent active surveillance, including 20 (16%) with visually [^18^F]FDG-negative nodules. To date (29 September 2021), the median follow-up is 29 months (IQR 24–45) and all nodules have remained unchanged on ultrasound: they are considered benign. All patients completed all study-related procedures. No patients were lost to follow-up.

**Table 1:** Baseline characteristics of included patients

	All (***n*** = 123)	Non-Hürthle cell, AUS/FLUS + FN/SFN^a^ **(*n* = 94)**	Hürthle cell (***n*** = 29)	*p*
	*n* (%)	*n* (%)	*n* (%)	
Female sex	102 (82.9%)	79 (84%)	23 (79%)	0.58^f^
Age (years) (mean ± SD)	55.0 ± 13.4	54.5 ± 13.0	55.7 ± 14.8	0.76^ g^
*Ultrasound characteristics*				
Solitary nodule	87 (71%)	64 (68%)	23 (79%)	0.25^f^
Dominant nodule in multinodular disease	36 (29%)	30 (32%)	6 (21%)	
Size (mm) (median, IQR)^b^	35 (22–44)	35 (22–44)	33 (23–43)	0.90^ h^
Suspicious characteristics^c^	49 (40%)	37 (39%)	12 (41%)	0.85^f^
Solid hypoechoic nodule	34 (28%)	27 (29%)	7 (24%)	0.63^f^
Taller-than-wide shape	1 (1%)	0 (0%)	1 (3%)	0.24^i^
Irregular margins	9 (7%)	8 (9%)	1 (3%)	0.68^i^
Microcalcifications	14 (11%)	10 (11%)	4 (14%)	0.74^i^
*Thyroid function*				
TSH, mU/L (median, IQR)^d^	1.70 (1.08–2.40)	1.70 (0.97–2.31)	1.70 (1.35–3.00)	0.25^ h^
fT4, pmol/L (median, IQR)^e^	14.6 (13.2–16.6)	14.6 (13.2–16.7)	14.2 (13.3–15.6)	0.62^ h^
Diagnostic surgery	100 (81.3%)	74 (79%)	26 (90%)	0.19^f^
*Malignant histopathology*	24 (20%)	18 (19%)	6 (21%)	0.86^f^
PTC	5	5	0	
FVPTC	4	4	0	
FTC, minimally invasive	6	6	0	
HCC, minimally invasive	5	0	5	
DTC not otherwise specified	1	0	1	
PDTC	1	1	0	
MTC	2	2	0	
*Borderline histopathology*	9 (7%)	6 (6%)	3 (10%)	0.44^i^
NIFTP	5	4	1	
FT-UMP, Hürthle cell type	3	1	2	
Paraganglioma	1	1	0	
*Benign histopathology*	67 (54%)	50 (53%)	17 (59%)	0.61^f^
Follicular adenoma	28	27	1	
Hürthle cell adenoma	13	4	9	
Hyperplastic nodule	26	19	7	
No surgery, unsuspicious on ultrasound f/u	23 (19%)	20 (21%)	3 (10%)	0.19^f^
[^18^F]FDG-positive	84 (68%)	56 (60%)	28 (97%)	** < 0.001**^** h**^
SUV_max_ nodule (g/mL) (median, IQR)^a^	4.0 (2.5–10.0)	3.4 (2.3–6.7)	12.3 (5.8–33.2)	** < 0.001**^** h**^
SUV_peak_ nodule (g/mL) (median, IQR)	3.3 (2.1–7.1)	2.8 (2.0–5.0)	12.3 (5.8–33.2)	** < 0.001**^** h**^
SUV_max_ thyroid background (g/mL) (median, IQR)	2.1 (1.2–5.2)	2.0 (1.8–2.5)	1.8 (1.6–2.2)	0.12^ h^
SUV_max_-ratio (median, IQR)	2.1 (1.2–5.2)	1.6 (1.1–2.8)	3.9 (6.4–12.1)	** < 0.001**^** h**^
SUV_peak_-ratio (median, IQR)	1.6 (1.0–5.2)	1.3 (0.9–2.2)	4.7 (2.8–10.7)	** < 0.001**^** h**^

*AUS/FLUS*, atypia of undetermined significance or follicular lesions of undetermined significance; *DTC*, differentiated thyroid carcinoma. *FN/SFN*, (suspicious for a) follicular neoplasm; *fT4*, free thyroxine; *FTC*, follicular thyroid carcinoma; *FT-UMP*, follicular tumour of uncertain malignant potential; *FVPTC*, follicular variant PTC; *HCC*, Hürthle cell carcinoma; *HCN/SHCN*, (suspicious for a) Hürthle cell neoplasm; *IQR*, interquartile range; *MTC*, medullary thyroid carcinoma; *PDTC*, poorly differentiated thyroid carcinoma; *PTC*, papillary thyroid carcinoma; *NIFTP*, non-invasive follicular thyroid neoplasm with papillary-like nuclear features; *SD*, standard deviation; *TSH*, thyroid stimulating hormone

^a^Baseline characteristics including SUV metrices were similar for AUS/FLUS (*n* = 55) and FN/SFN (*n* = 39) subgroups; the baseline data of these subgroups are presented in the Supplementary table 4

^b^In all patients, ultrasound nodule size was not correlated with the SUV_max_ (*r*(121) = 0.13, *p* = 0.154)

^c^Suspicious ultrasound characteristics were defined as presence of at least one of the following characteristics: marked hypoechogenicity (in a solid nodule), irregular shape (i.e., taller-than-wide), irregular margins, and/or presence of microcalcifications

^d^The reference range for TSH is 0.4–4.0 mU/L

^e^The reference range for fT4 is approximately 10–25 pmol/L (sex and age dependent)

^f^Pearson’s chi-squared test

^g^Independent samples *t*-test

^h^Mann-Whitney *U* test

^i^Fisher’s exact test

### Visual [^18^F]FDG-PET/CT assessment

Thirty-one of 33 (94%) malignant/borderline nodules and 53 of 90 (59%) benign nodules were visually [^18^F]FDG-positive. The median SUV_max_ was 7.1 g/mL (IQR, 3.9–13.9) in [^18^F]FDG-positive and 2.3 g/mL (IQR, 1.9–2.8) in [^18^F]FDG-negative nodules. Two low-risk malignancies (one pT1bN0 and one pT2N0) were [^18^F]FDG-negative (false-negative). The diagnostic accuracy of visual [^18^F]FDG-PET/CT assessment in the current study was similar to the original trial (*n* = 132) (Table 2) [4]. All but one of 29 Hürthle cell nodules were visually [^18^F]FDG-positive, resulting in a 3.4% benign call rate (Table 2).

**Table 2:** Threshold analysis and diagnostic accuracy

	**SUV cut-off**	**TP**	**FP**	**TN**	**FN**	**Sensitivity, % (95% CI)**	**Specificity, % (95% CI)**	**NPV, % (95% CI)**	**PPV, % (95% CI)**	**Benign call rate, % (95% CI)**
**Visual assessment**										
All (*n* = 123)		31	53	37	2	93.9 (79.8–99.3)	41.1 (30.8–52.0)	94.9 (82.7–99.4)	36.9 (26.6–48.1)	31.7 (23.6–40.7)
Non-Hürthle cell nodules (*n* = 94)		22	34	36	2	91.7 (73.0–99.0)	51.4 (39.2–63.6)	94.7 (82.3–99.4)	39.3 (26.5–53.2)	40.4 (30.4–51.0)
Hürthle cell nodules (*n* = 29)		9	19	1	0	100 (66.4–100)	5.0 (0.1–24.9)	100 (2.5–100)	32.1 (15.9–52.4)	3.4 (0.1–17.8)
**Quantitative analysis**										
**All (*n* = 123)**										
SUV_max_ nodule, g/mL	2.1	32	73	17	1	97.0 (84.2–99.9)	18.9 (11.4–28.5)	94.4 (72.7–99.9)	30.5 (21.9–40.2)	14.6 (8.9–22.1)
SUV_peak_ nodule, g/mL	1.6	32	80	10	1	97.0 (84.2–99.9)	11.1 (5.5–19.5)	90.9 (58.7–99.8)	28.6 (20.4–37.9)	8.9 (4.5–15.4)
SUV_max_-ratio	1.2	32	56	34	1	97.0 (84.2–99.9)	37.8 (27.8–48.6)	97.1 (85.1–99.9)	36.4 (26.4–47.3)	28.5 (20.7–37.3)
SUV_peak_-ratio	0.9	32	75	15	1	97.0 (84.2–99.9)	16.7 (9.6–26.0)	93.8 (69.8–99.8)	29.9 (21.4–39.5)	13.0 (7.6–20.3)
**Non-Hürthle cell nodules, AUS/FLUS + FN/SFN**^**a**^** (*n* = 94)**										
SUV_max_ nodule, g/mL	2.1	23	54	16	1	95.8 (78.9–99.9)	22.9 (13.7–34.4)	94.1 (71.3–99.9)	29.9 (20.0–41.4)	18.1 (10.9–27.4)
SUV_peak_ nodule, g/mL	1.6	23	61	9	1	95.8 (78.9–99.9)	12.9 (6.1–23.0)	90.0 (55.5–99.7)	27.4 (18.2–38.2)	10.6 (5.2–18.7)
SUV_max_-ratio	1.2	23	38	32	1	95.8 (78.9–99.9)	45.7 (33.7–58.1)	97.0 (84.2–99.9)	37.7 (25.6–51.0)	35.1 (25.5–45.6)
SUV_peak_-ratio	0.9	23	57	13	1	95.8 (78.9–99.9)	18.6 (10.3–29.7)	92.9 (66.1–99.8)	28.8 (19.2–40.0)	14.9 (8.4–23.7)
**Hürthle cell nodules, HCN/SHCN (*n* = 29)**										
SUV_max_ nodule, g/mL	5.2	9	15	5	0	100 (66.4–100)	25.0 (8.7–49.1)	100 (47.8–100)	37.5 (18.8–59.4)	17.2 (5.8–35.8)
SUV_peak_ nodule, g/mL	4.7	9	13	7	0	100 (66.4–100)	35.0 (15.4–59.2)	100 (59.0–100)	40.9 (20.7–63.6)	24.1 (10.3–43.5)
SUV_max_-ratio	3.4	9	14	6	0	100 (66.4–100)	30.0 (11.9–54.3)	100 (54.1–100)	39.1 (19.7–61.5)	20.7 (8.0–39.7)
SUV_peak_-ratio	2.8	9	13	7	0	100 (66.4–100)	35.0 (15.4–59.2)	100 (59.0–100)	40.9 (20.7–63.6)	24.1 (10.3–43.5)

*AUS/FLUS*, atypia of undetermined significance or follicular lesions of undetermined significance; *CI*, confidence interval; *FN*, false negative; *FN/SFN*, (suspicious for a) follicular neoplasm; *FP*, false positive; *HCN/SHCN*, (suspicious for a) Hürthle cell neoplasm; *NPV*, negative predictive value; *PPV*, positive predictive value; *SUV*, standardised uptake value; *TN*, true negative; *TP*, true positive

^a^SUV cut-offs were similar for AUS/FLUS (*n* = 55) and FN/SFN (*n* = 39) subgroups; results for these subgroups are presented in the Supplementary table 7

### Quantitative [^18^F]FDG-PET/CT assessment

**Fig. 3:**
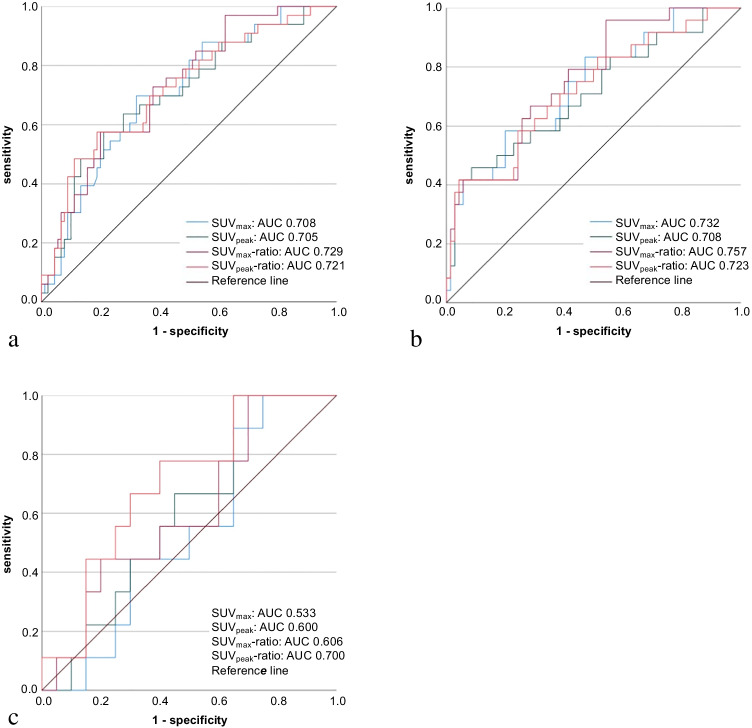
ROC curves of quantitative [^18^F]FDG-PET/CT analysis. ROC curves for SUV_max_ (blue line), SUV_peak_ (green), SUV_max_-ratio (purple), and SUV_peak_-ratio (red) in **a** all (*n* = 123), **b** non-Hürthle cell (*n* = 94), and **c** Hürthle cell (*n* = 29) nodules. **a: **In all nodules, the AUCs for the SUV_max_, SUV_peak_, SUV_max_-ratio, and SUV_peak_-ratio were 0.708 (95% CI, 0.609–0.807), 0.705 (0.601–0.810), 0.729 (0.633–0.824), and 0.721 (0.618–0.824), respectively. **b:** In non-Hürthle cell nodules, these AUCs were 0.732 (95% CI, 0.615–0.849), 0.708 (0.580–0.835), 0.757 (0.650–0.864), and 0.723 (0.601–0.844), respectively. **c:** In Hürthle cell nodules, these AUCs were 0.533 (95% CI, 0.320–0.747), 0.600 (0.392–0.808), 0.606 (0.388–0.823), and 0.700 (0.502–0.898), respectively

In all 123 nodules, the median SUV_max_, SUV_peak_, SUV_max_-ratio, and SUV_peak_-ratio were significantly higher in malignant/borderline nodules than in benign nodules (*p* < 0.001) (Table 3). ROC curve analysis showed similar AUCs for all SUV-metrices (Fig. 3). A 97.0% sensitivity was reached at SUV_max_, SUV_peak_, SUV_max_-ratio, and SUV_peak_-ratio cut-offs of 2.1 g/mL, 1.6 g/mL, 1.2, and 0.9, respectively (Table 2). At these cut-offs, the benign call rate varied between 8.9% for the SUV_peak_ and 28.5% for the SUV_max_-ratio. Missed malignant/borderline tumours varied across the SUV-metrices and included the two visually false-negative nodules and a 20 mm NIFTP with a SUV_max_ of 2.1 g/mL and SUV_peak_ of 1.6 g/mL.

**Table 3:** Differences in SUV metrices between malignant/borderline and benign nodules

	Malignant/borderline	Benign	*p*
**All (*n* = 123)**	***n* = 33**	***n* = 90**	
SUV_max_ nodule, g/mL	8.3 (3.6–16.3)	3.4 (2.3–7.3)	** < 0.001**
SUV_peak_ nodule, g/mL	6.1 (2.8–12.6)	2.9 (1.9–5.6)	** < 0.001**
SUV_max_ thyroid background, g/mL	1.8 (1.7–2.2)	2.0 (1.8–2.5)	0.17
SUV_max_-ratio	4.0 (1.9–8.8)	1.7 (1.1–3.2)	** < 0.001**
SUV_peak_ ratio	3.3 (0.9–2.5)	1.3 (0.9–2.5)	** < 0.001**
**Non-Hürthle cell nodules,**			
**AUS/FLUS + FN/SFN**^**a**^** (*n* = 94)**	***n* = 24**	***n* = 70**	
SUV_max_ nodule, g/mL	5.8 (3.3–15.2)	3.1 (2.3–4.7)	** < 0.001**
SUV_peak_ nodule, g/mL	4.5 (2.5–10.9)	2.5 (1.9–3.9)	**0.002**
SUV_max_ thyroid background, g/mL	1.9 (1.7–2.4)	2.0 (1.8–2.5)	0.22
SUV_max_-ratio	2.5 (1.6–8.1)	1.5 (1.0–2.5)	** < 0.001**
SUV_peak_-ratio	2.1 (1.2–5.3)	1.2 (0.9–1.9)	**0.001**
**Hürthle cell nodules,**			
**HCN/SHCN (*n* = 29)**	***n* = 9**	***n* = 20**	
SUV_max_ nodule, g/mL	12.3 (8.0–28.4)	12.2 (5.0–35.3)	0.80
SUV_peak_ nodule, g/mL	9.9 (6.1–24.0)	7.3 (4.0–22.5)	0.42
SUV_max_ thyroid background, g/mL	1.8 (1.6–2.1)	1.9 (1.6–2.5)	0.66
SUV_max_-ratio	7.4 (4.7–14.3)	6.3 (2.7–10.9)	0.39
SUV_peak_-ratio	5.9 (3.8–11.4)	4.1 (2.2–8.3)	0.10

*AUS/FLUS*, atypia of undetermined significance or follicular lesions of undetermined significance; *CI*, confidence interval; *FN/SFN*, (suspicious for a) follicular neoplasm; *HCN/SHCN*, (suspicious for a) Hürthle cell neoplasm; *SUV*, standardised uptake value. SUV values are presented as median (IQR) and compared between groups using the Mann–Whitney *U* test

^a^: Results for AUS/FLUS (n = 55) and FN/SFN (n = 39) subgroups are presented in the Supplementary table 6

In the 94 non-Hürthle cell nodules, a sensitivity of 95.8% was established at the same cut-offs, with the benign call rate ranging from 10.6 to 35.1% (Table 2). Similar cut-offs for all SUV metrices and similar benign call rates were found in AUS/FLUS as compared to FN/SFN nodules (Supplementary tables 6–7, Supplementary Fig. 1). In the 29 Hürthle cell nodules, no significant differences in SUV-metrices were found between malignant/borderline and benign nodules (Table 3). The AUCs ranged from 0.533 for the SUV_max_ to 0.700 for the SUV_peak_-ratio (Fig. 3). Yet, at SUV cut-offs of 5.2 g/mL, 4.7 g/mL, 3.4, and 2.8, sensitivity was 100% with benign call rates ranging from 17.2% for the SUV_max_ to 24.1% for the SUV_peak_ and SUV_peak_-ratio (Table 2).

Subgroup analysis of the visually [^18^F]FDG-positive non-Hürthle cell nodules showed similar SUV values in malignant/borderline and benign nodules. Threshold analysis in this subgroup showed that a ≥ 95% sensitivity was only achieved at minimal benign call rates (Supplementary table 8 and 9).

### Radiomic analysis

**Fig. 4:**
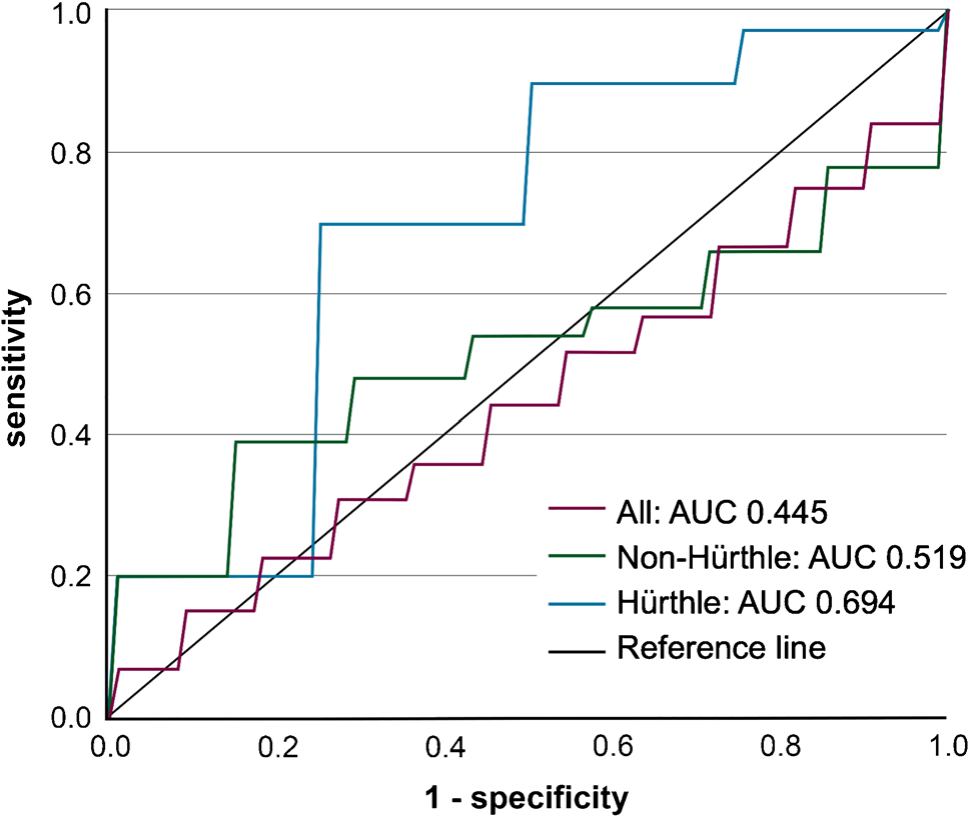
ROC curves of the PET model of the radiomic analysis. ROC curves for the PET/CT model of the radiomic analysis. The AUC was 0.445 (95% CI, 0.290–0.600) in all nodules (*n* = 84, purple line), 0.519 (95% CI, 0.298–0.740) in non-Hürthle cell nodules (*n* = 56, green), and 0.694 (95% CI, 0.461–0.926) in Hürthle cell nodules (*n* = 28, blue)

The 84 (68%) patients with visually [^18^F]FDG-positive nodules were included in the radiomic analysis, including 56 (67%) non-Hürthle and 28 (33%) Hürthle cell nodules (Fig. 1). Dimensionality reduction of the radiomic feature set retained six factors in every training set (68 patients in training sets). The mean AUC of the PET model was 0.445 in the test set (Fig. 4, Supplementary table 10). The retained factors corresponded to entropy of the intensity histogram, nodule size, high intensity on PET, variance in area size, total lesions glycolysis, and small areas with low grey levels. KMOs in all folds were excellent (≥ 0.927). Subgroup analyses in non-Hürthle cell and Hürthle cell nodules resulted in test set AUCs for the PET models of 0.519 and 0.694, respectively (Supplementary table 10). The performances of the PET/CT models were similar, indicating limited diagnostic accuracy (Supplementary table 10, Supplementary Fig. 2). Subgroup analysis for nodules meeting the minimal size recommendation of 64 voxels per VOI (*n* = 66) demonstrated a similar performance with an AUC of 0.421 for the PET model (Supplementary table 10) [35].

## Discussion

The *EfFECTS* trial showed that *visual* assessment of [^18^F]FDG PET/CT had a practice-changing rule-out ability in Bethesda III/IV nodules. In non-Hürthle cell nodules, [^18^F]FDG-PET/CT-driven management accurately avoided nearly half of the futile diagnostic surgeries for benign nodules. It was not contributing, however, in the nearly exclusively strongly [^18^F]FDG-positive Hürthle cell nodules [4]. In the current side study of this randomised controlled trial, we showed that *quantitative* [^18^F]FDG-PET/CT analysis accurately ruled out malignancy in both non-Hürthle and Hürthle cell nodules, provided that different SUV cut-offs were chosen for these two groups. In Hürthle cell nodules, relatively high SUV cut-offs resulted in an excellent sensitivity and benign call rates up to 24%. Consequently, a maximum 35% (7 of 20) of diagnostic surgeries could have been avoided for benign Hürthle cell nodules in the current cohort. Even though the reported SUV cut-offs require external validation in future prospective studies prior to implementation in clinical practice, quantitative assessment thus appears to have a major advantage over visual assessment in Hürthle cell nodules. In non-Hürthle cell nodules, an excellent rule-out ability was demonstrated at relatively low SUV cut-offs with moderate (11%) to excellent (35%) benign call rates. As a result, a maximum of 46% (32 of 70) surgeries for benign nodules could have been avoided in AUS/FLUS and FN/SFN nodules. These results were similar to our previous findings regarding the visual interpretation of [^18^F]FDG-PET/CT [4]. Unfortunately, specificity was similarly limited, too: many benign nodules were still considered false-positive on quantitative assessment. Additional subgroup analysis showed that quantitative [^18^F]FDG-PET/CT assessment lacked discriminative capacity in visually [^18^F]FDG-positive non-Hürthle cell nodules. In contrast to Hürthle cell nodules, there were no separate (higher) SUV cut-offs that contributed to a better differentiation for this subgroup. As such, quantitative [^18^F]FDG-PET/CT assessment appeared to have no additional diagnostic value over visual assessment in non-Hürthle cell nodules, and is likely best applied to support the visual assessment [4].

Radiomic analysis on PET/CT did not contribute to the differentiation of [^18^F]FDG-positive non-Hürthle cell and/or Hürthle cell nodules, with AUCs ranging from 0.445 to 0.694.

**Fig. 5:**
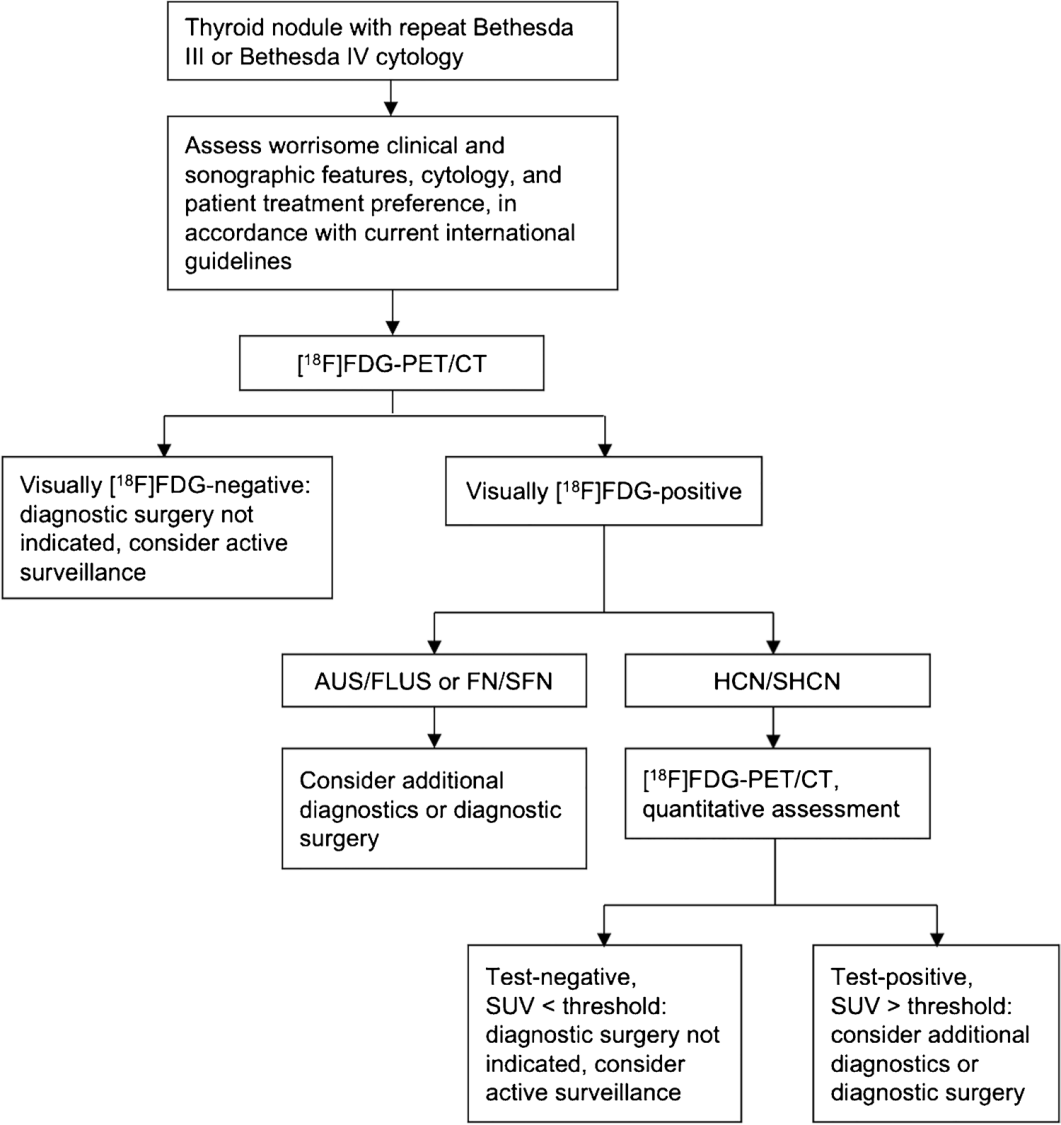
Proposed [^18^F]FDG-PET/CT-driven workup of Bethesda III/IV thyroid nodules

Based on the results of our previous and the current study, we suggest a diagnostic algorithm for the [^18^F]FDG-PET/CT-driven workup of Bethesda III and IV thyroid nodules (Fig. 5). If externally validated, this workup could prevent more than half of the futile diagnostic surgeries for benign nodules. Additional diagnostics could be considered to further improve the differentiation of [^18^F]FDG-*positive* non-Hürthle cell nodules and Hürthle cell nodules, including molecular diagnostics and systematic ultrasound evaluation using the Thyroid Imaging Reporting and Data System (TIRADS) [16]. Combined [^18^F]FDG-PET/CT and TIRADS assessment previously showed high diagnostic accuracy in indeterminate thyroid nodules [12]. The performance of EU-TIRADS in Hürthle cell nodules seems more limited [37].

The limited number of prior studies on quantitative [^18^F]FDG-PET/CT assessment in indeterminate thyroid nodules reported major variations in SUV cut-offs and diagnostic accuracy. Deandreis et al. and Rosario et al., who respectively included 56 indeterminate nodules (including 29 [52%] with Hürthle cell cytology) and 63 Bethesda III/IV nodules, showed that a SUV_max_ of at least 5 g/mL was 91% specific to detect thyroid carcinoma, NIFTP, and FT-UMP [6, 10]. In contrast, Merten et al. found that the same cut-off was only 41% specific but 80% sensitive in their study in 51 Bethesda IV nodules (including 24 [47%] Hürthle cell cytology) [8]. Piccardo et al. reported that a SUV_max_-*ratio* of 5 was the most accurate, without reporting an AUC or corresponding sensitivity and specificity in 111 indeterminate nodules [12]. Pathak et al. excluded Hürthle cell nodules and reported that a SUV_max_ cut-off of 3.25 g/mL best differentiated the remaining 42 non-Hürthle cell nodules with 79% sensitivity and 83% specificity [38]. Part of the mixed results of these studies may be explained by different compositions of the patient populations, including the fractions of Hürthle cell cytology. Unfortunately, none of these studies separately analysed non-Hürthle and Hürthle cell nodules, even though multiple studies have reported higher [^18^F]FDG uptake in Hürthle cell nodules and it has repeatedly been suggested that Hürthle cell nodules should be treated as separate entities in the diagnostic workup [7, 14]. Besides that, SUV calculations strongly depend, amongst others, on image acquisition and reconstruction settings, and PET-scanner model [7, 16]. It requires harmonised [^18^F]FDG-PET protocols to enable the global inter-institution comparison of study results and advancement of PET research [26, 39].

None of these previous studies used ROC curve analysis to determine SUV cut-offs that corresponded to optimal test sensitivity, even though threshold analysis seems a suitable method to uphold the ATA recommendations for a useful additional diagnostic (i.e., ≥ 96% NPV for a rule-out test) [25].

To the best of our knowledge, our study is the second to report PET/CT radiomics in indeterminate thyroid nodules. Giovanella et al. recently published the first study in 78 Bethesda III/IV patients, demonstrating a 96% NPV and 58% PPV for a multiparametric model including the cytological classification and two radiomic features [19]. PPV improved to 79% if 13 patients with a histopathological Hürthle cell adenoma were excluded (cytology not reported). Supervised feature selection was performed using redundancy filtering of features strongly correlating to SUV_max_ and the metabolic tumour volume (*ρ* > 0.7) and LASSO logistic regression. The included features were GLCM autocorrelation and shape sphericity. In our factor-based analysis, the feature GLCM autocorrelation was frequently the underlying factor for ‘high intensity on PET’, a factor that was also often accompanied by SUV_max_. Shape sphericity was not one of the main features that explained our factors. Giovanella et al.’s radiomic models resulted in AUCs of 0.73 in all nodules and in non-Hürthle cell nodules. Despite different radiomic methodology, Giovanella et al. also concluded that sole radiomic analysis on [^18^F]FDG-PET/CT provides no added value in the workup of indeterminate thyroid nodules. When incorporated in the proposed multiparametric model, however, clinical application of radiomics seems feasible. Future studies are required to validate their results [19].

One of the strengths of our study is its carefully evaluated radiomic methodology. First, we preferred unsupervised feature selection or dimensionality reduction over supervised feature selection, which uses discriminative values for the outcome. Unsupervised methods take into account the interaction of features and multicollinearity, thereby preventing overfitting of the model [40]. We selected non-redundant features with low multicollinearity, which were not necessarily the features with the highest predictive performance. Second, dimensionality reduction was performed on the training sets in the folds instead of on the dataset as a whole, strictly distinguishing the independent test sets. Third, factor-based dimensionality reduction was chosen over a feature-based approach for generalizability purposes. Instead of selecting features corresponding to the retained factors, the factors were used as input for the model and patterns in corresponding features were compared between folds. In a feature-based approach, different features might have been selected in different folds, resulting in limited insight in these patterns. Along these lines, a factor-based approach improves the generalizability and interpretability of the model and might provide insight in the semantics or underlying tumour biology of the factors [41]. Contrarily, it reduces the (mathematical) explainability and reproducibility of the radiomic model during external validation, as it uses derivatives of features. Adherence to the IBSI reporting guidelines and TRIPOD statement may prevent reproducibility issues [31, 36]. Another limitation is that eighteen nodules did not meet the minimal size recommendation for radiomic analysis of 64 voxels per VOI [35]. Subgroup analysis of the nodules meeting this requirement showed similar results. It is unlikely that the nodule size had a large impact on the radiomic analysis.

The multicentre design of the study was both a strength and limitation. While the population of our nationwide trial is unique and an adequate reflection of the diverse presentation of thyroid nodules, the different scanners and slight variations in imaging protocols among the 12 hospitals introduced heterogeneity and may have limited the radiomic analysis. Therefore, only scans with strict adherence to the EANM guidelines were assessed, as these reconstructions leads to a larger number of reliable, repeatable, and reproducible radiomic features in a multicentre and multivendor setting [42]. In addition, nodules were delineated using a threshold of 50% of the SUV_peak_, corrected for local background, which is recommended in multicentre [^18^F]FDG PET/CT studies because of its high feasibility and repeatability [29]. Moreover, all images were interpolated to isotropic voxels in order to allow comparison between image data from different samples and centres [43]. The number of included patients per centre or PET/CT-scanner was not sufficiently large to incorporate post-reconstruction harmonization strategies such as ComBat [44].

In conclusion, the current study showed that quantitative [^18^F]FDG-PET/CT assessment accurately ruled out malignancy in both Hürthle cell and non-Hürthle cell indeterminate thyroid nodules. Distinctive SUV cut-offs may avoid up to one in three futile diagnostic surgeries for benign Hürthle cell nodules. In non-Hürthle cell nodules, quantitative assessment had no added diagnostic value over visual [^18^F]FDG-PET/CT assessment. Radiomic analysis did not contribute to the additional differentiation of [^18^F]FDG-positive thyroid nodules in this dataset.

## Supplementary Information

Below is the link to the electronic supplementary material.

Supplementary file1 (DOCX 2140 KB)

## Data Availability

The datasets and models generated during and/or analysed during the current study are available from the corresponding author on reasonable request.
