# Supplementary material for: Quantitative classification and radiomics of [18F]FDG-PET/CT in indeterminate thyroid nodules
Source: Eur J Nucl Med Mol Imaging. 2022 Feb 9;49(7):2174–88. doi: 10.1007/s00259-022-05712-0 (PMC9165273; doi:10.1007/s00259-022-05712-0)
Supplement: Supplementary file 1 — Supplementary file1 (DOCX 2140 KB) [file 259_2022_5712_MOESM1_ESM.docx]

**Supplementary Data**

**Quantitative classification and radiomics of [^18^F]FDG-PET/CT**

**in indeterminate thyroid nodules**

# Elizabeth J. de Koster, Wyanne A. Noortman, Jacob M. Mostert, Jan Booij, Catherine B. Brouwer, Bart de Keizer, John M.H. de Klerk, Wim J.G. Oyen, Floris H.P. van Velden, Lioe-Fee de Geus-Oei, Dennis Vriens

for the *EfFECTS trial* study group.

**Table of contents**

*EfFECTS trial* study group 3.

Trial steering committee 3.

Local principal investigators 3.

Study safety committee 4.

Supplementary table 1: Included patients per study site and PET/CT scanner 5.

Supplementary table 2: Image Biomarker Standardisation Initiative (IBSI) Reporting Guidelines 6.

Supplementary table 3: Baseline characteristics of included as compared to excluded patients 12.

Supplementary table 4: Baseline characteristics of included patients (3 groups) 13.

Supplementary table 5: Baseline characteristics of malignant/borderline nodules as compared 15.

to benign nodules

Supplementary table 6: Differences in SUV metrices between malignant/borderline and benign 16.

nodules in AUS/FLUS and FN/SFN nodules

Supplementary table 7: Threshold analysis and diagnostic accuracy for AUS/FLUS and 17.

FN/SFN nodules

Supplementary figure 1: ROC curves of quantitative [^18^F]FDG-PET/CT analysis in 18.

AUS/FLUS and FN/SFN nodules

Supplementary table 8: Results of ROC curve analysis in [^18^F]FDG-positive non-Hürthle 19.

cell nodules (n=56)

Supplementary table 9: Threshold analysis and diagnostic accuracy in [^18^F]FDG-positive 20.

non-Hürthle cell nodules (n=56)

Supplementary table 10: Radiomic analysis: dimensionality reduction and predictive 21.

performance of radiomic models for all [18F]FDG-positive nodules, non-Hürthle cell nodules and Hürthle cell nodules.

Supplementary figure 2: ROC curves of the radiomic analysis, PET/CT model 22.

References 23.

**EfFECTS trial study group**

**Trail steering committee**

- Prof. dr. L.F. de Geus-Oei, MD PhD (**project leader**), *Leiden University Medical Centre, Department of Radiology, Section of Nuclear Medicine, Leiden, the Netherlands; Radboud University Medical Centre, Department of Radiology and Nuclear Medicine, Nijmegen, the Netherlands*
- Prof. dr. W.J.G. Oyen, MD PhD (**principal investigator**), *Radboud University Medical Centre, Department of Radiology and Nuclear Medicine, Nijmegen, the Netherlands; Rijnstate Hospital, Department of Radiology and Nuclear Medicine, Arnhem, the Netherlands; Department of Biomedical Sciences and Humanitas Clinical and Research Centre, Department of Nuclear Medicine, Humanitas University, Milan, Italy*
- Dr. D. Vriens, MD PhD (**principal investigator**), *Leiden University Medical Centre, Department of Radiology, Section of Nuclear Medicine, Leiden, the Netherland.*
- E.J. de Koster, MD (**junior investigator**), *Radboud University Medical Centre, Department of Radiology and Nuclear Medicine, Nijmegen, the Netherlands*

**Local principal investigators**

**Radboud university medical centre, Nijmegen, the Netherlands**

- Dr. A.C.H. van Engen-van Grunsven, MD PhD, *Department of Pathology*
- E.J. de Koster, MD, *Department of Radiology and Nuclear Medicine*
- Dr. B. Kusters, MD PhD, *Department of Pathology*
- Prof. dr. R.T. Netea-Maier, MD PhD, *Department of Internal Medicine, Division of Endocrinology*
- Prof. dr. J.W.A. Smit, MD PhD, *Department of Internal Medicine, Division of Endocrinology*
- Prof. dr. J.H.W. de Wilt, MD PhD, *Department of Surgical Oncology*

**Amsterdam University Medical Centre, Amsterdam, the Netherlands**

*Location AMC*

- Prof. dr. J. Booij, MD PhD, *Department of Radiology and Nuclear Medicine*
- Prof. dr. E. Fliers, MD PhD, *Department of Endocrinology and Metabolism*
- Dr. T.K. Klooker, MD PhD, *Department of Endocrinology and Metabolism*

*Location VUMC*

- Dr. E.W.C.M. van Dam, MD PhD, *Department of Internal Medicine, Division of Endocrinology*
- Dr. K.M.A. Dreijerink, MD PhD, *Department of Internal Medicine, Division of Endocrinology*
- Dr. P.G.H.M. Raijmakers, MD PhD, *Department of Radiology and Nuclear Medicine*

**Erasmus University Medical Centre, Rotterdam, the Netherlands**

- Dr. B.L.R. Kam, MD PhD, *Department of Nuclear Medicine*
- Prof. dr. R.P. Peeters, MD PhD, *Department of Internal Medicine*
- Prof. dr. J. Verzijlbergen, MD PhD, *Department of Nuclear Medicine*

**Haga Hospital, The Hague, the Netherlands**

- Dr. M.O. van Aken, MD PhD, *Department of Internal Medicine*

**Isala Hospital, Zwolle, the Netherlands**

- Prof. dr. P.L. Jager, MD PhD, *Department of Nuclear Medicine*
- Dr. G.S. Mijnhout, MD PhD, *Department of Internal Medicine*

**Leiden University Medical Centre, Leiden, the Netherlands**

- Prof. dr. L.F. de Geus-Oei, MD PhD, *Department of Radiology, Section of Nuclear Medicine*
- Dr. W.B. van den Hout, PhD, *Department of Biomedical Data Sciences-Medical Decision Making*
- Prof. dr. A.M. Pereira Arias, MD PhD, *Department of Internal Medicine, Division of Endocrinology*
- Prof. dr. J. Morreau, MD PhD, *Department of Pathology*
- Dr. M. Snel, MD PhD, *Department of Internal Medicine, Division of Endocrinology*
- Dr. D. Vriens, MD PhD, *Department of Radiology, Section of Nuclear Medicine*

**Meander Medical Centre, Amersfoort, the Netherlands**

- Dr. L.T. Dijkhorst-Oei, MD PhD, *Department of Internal Medicine*
- Dr. J.M.H. de Klerk, MD PhD, *Department of Nuclear Medicine*

**Maastricht University Medical Centre, Maastricht, the Netherlands**

- Dr. B. Havekes, MD PhD, *Department of Internal Medicine, Division of Endocrinology*
- Dr. D.C. Mitea, MD PhD, *Department of Radiology and Nuclear Medicine*
- Dr. S. Vöö, MD PhD, *Department of Radiology and Nuclear Medicine*

**OLVG Hospital, Amsterdam, the Netherlands**

- Dr. C.B. Brouwer, MD PhD, *Department of Internal Medicine*
- Dr. P.S. van Dam, MD PhD, *Department of Internal Medicine*
- Dr. F. Sivro, MD PhD, *Department of Nuclear Medicine*

**Reinier de Graaf Hospital, Delft, the Netherlands**

- Dr. E.T. te Beek, MD PhD, *Department of Nuclear Medicine*
- Dr. M.C.W. Jebbink, MD PhD, *Department of Internal Medicine*

**Rijnstate Hospital, Arnhem, the Netherlands**

- Dr. G.S. Bleumink, MD PhD, *Department of Internal Medicine*
- Prof. dr. W.J.G. Oyen, MD PhD, Department of Radiology and Nuclear Medicine
- Dr. V.J.R. Schelfhout, MD PhD, Department of Radiology and Nuclear Medicine

**St. Antonius Hospital, Nieuwegein, the Netherlands**

- Dr. R.G.M. Keijsers, MD PhD, *Department of Nuclear Medicine*
- Dr. I.M.M.J. Wakelkamp, MD PhD, *Department of Internal Medicine*

**University Medical Centre Groningen, Groningen, the Netherlands**

- Dr. A.H. Brouwers, MD PhD, *Department of Nuclear Medicine and Molecular Imaging*
- Prof. dr. T.P. Links, MD PhD, *Division of Endocrinology, Department of Internal Medicine*

**University Medical Centre Utrecht, Utrecht, the Netherlands**

- Dr. B. de Keizer, MD PhD, *Department of Radiology and Nuclear Medicine*
- Dr. R. van Leeuwaarde, MD PhD, *Department of Endocrine Oncology*

**Study safety committee**

- Dr. J.J. Bonenkamp, MD PhD, *Department of Surgical Oncology, Radboud University Medical Centre, Nijmegen, The Netherlands.*
- Dr. A.R.T. Donders, PhD, *Department for Health Evidence, Radboud University Medical Centre, Nijmegen, the Netherlands*
- Prof. dr. J.J. Fütterer, Phd, *Department of Radiology and Nuclear Medicine, Radboud University Medical Centre, Nijmegen, The Netherlands.*

**Supplementary table 1:**

**Included patients per study site and PET/CT scanner**

| **Study site** | **Included patients at study site** | **PET/CT scanners** | **patients per PET/CT scanner** | **patients per PET/CT scanner, included in radiomic analysis** |
| --- | --- | --- | --- | --- |
| Radboud university medical centre, Nijmegen, the Netherlands | 13 | Siemens SOMATOM Definition AS | 2 | 2 |
|  |  | Siemens Biograph mCT 40 | 10 | 8 |
|  |  | Philips Gemini TF 64 | 1 | 0 |
| Amsterdam University Medical Centre, Amsterdam, the Netherlands  Location AMC | 11 | Philips Gemini TF 16 | 5 + 1^a^ | 3 + 1^a^ |
|  |  | Philips Gemini GXL | 0 + 1^a^ | 0 + 1^a^ |
|  |  | Siemens Biograph mCT 128 | 6 + 4^a^ | 5 + 4^a^ |
| Amsterdam University Medical Centre, Amsterdam, the Netherlands  Location VUMC | 23 | Philips Ingenuity TF | 21 | 11 |
|  |  | Philips Gemini TF 64 | 2 | 1 |
| Erasmus University Medical Centre, Rotterdam, the Netherlands | 7 | Siemens Biograph mCT 40 | 7 | 5 |
| Haga Hospital, The Hague, the Netherlands | 4 | Patients were scanned at Leiden University Medical Center | n.a. | n.a. |
| Isala Hospital, Zwolle, the Netherlands | 5 | Philips Ingenuity TF | 5 | 2 |
| Leiden University Medical Center, Leiden, the Netherlands | 19 | Philips Gemini TF 64 | 10 + 2^b^ | 8 + 2^b^ |
|  |  | Siemens Biograph Horizon | 8 + 1^b^ | 4 + 1^b^ |
|  |  | Philips Vereos | 1 + 1^b^ | 1 + 1^b^ |
| Meander Medical Centre, Amersfoort, the Netherlands | 18 | Siemens Biograph mCT 40 | 18 | 9 |
| Maastricht University Medical Centre, Maastricht, the Netherlands | 2 | Philips Gemini TF 64 | 2 | 2 |
| OLVG Hospital, Amsterdam, the Netherlands | 6 | Patients were scanned at Amsterdam University Medical Center, location AMC | n.a. | n.a. |
| Reinier de Graaf Hospital, Delft, the Netherlands | 0 | n.a. | n.a. | n.a. |
| Rijnstate Hospital, Arnhem, the Netherlands | 2 | Philips Gemini TF 64 | 2 | 0 |
| St. Antonius Hospital, Nieuwegein, the Netherlands | 3 | Philips Gemini TF 64 | 3 | 2 |
| University Medical Centre Groningen, Groningen, the Netherlands | 14 | Siemens Biograph mCT 40 | 5 | 2 |
|  |  | Siemens Biograph mCT 64 | 9 | 7 |
| University Medical Centre Utrecht, Utrecht, the Netherlands | 5 | Siemens Biograph mCT 40 | 5 | 2 |
| **TOTAL** | **132** |  | **132** | **84** |

n.a., not applicable. ^a^: patients from OLVG Hospital. ^b^: patients from Haga Hospital.

**Supplementary table 2:**

**Image Biomarker Standardisation Initiative (IBSI) Reporting Guidelines [1]**

| **Patient** | | |
| --- | --- | --- |
| Volumes of interest | [^18^F]FDG-positive thyroid nodules with indeterminate cytology (Bethesda III / IV cytology) | |
| Patient preparation   - Patient instructions - Drugs - Equipment | Patients were advised to fast for at least 6 hours before imaging.  Only patients who fasted for at least 4 hours were included in the study [2]. | |
| Radioactive tracer   - Tracer - Administration method - Injected activity - Uptake time prior to acquisition - Competing substances | PET acquisition was started 60 (55-75) minutes after intravenous administration of [^18^F]FDG (3 MBq/kg of body weight) in a peripheral vein. Serum glucose levels were between 3.9 and 9.5 mmol/L (study protocol: <11.1 mmol/L) | |
| Contrast agent | NA | |
| Comorbidities | Patients with diabetes mellitus were allowed as long as serum glucose was below 11.1 mmol/L and no short-acting insulins were given 4 hours prior to injection of [^18^F]FDG.  All patients were euthyroid, in case of suppressed TSH autonomy was excluded by thyroid scintigraphy prior to inclusion (n=4).  Patients with evidence of infection localised to the neck in the 14 days prior to PET/CT would be ineligible for inclusion (n=0). | |
| **Acquisition** | | |
| Acquisition protocol | A low-dose CT (ldCT) was performed, followed by a static PET scan, from the meatus acusticus externus to the fossa jugularis sternalis (or down to the lower margin of the arcus aortae in case of retrosternal goitre) approximately 60 minutes post-injection. | |
| Scanner type | Scans were acquired at 12 study sites on 20 different PET/CT systems, using the following scanners:  Philips: Gemini-GXL, Gemini TF 16, Gemini TF 64, Ingenuity TF, Vereos (Philips Healthcare, Best, the Netherlands)  Siemens: Biograph mCT 40, Biograph mCT 64, Biograph mCT 128, Biograph Horizon, SOMATOM Definition AS (Siemens Healthineers, Erlangen, Germany) | |
| Imaging modality | PET/CT | |
| Static/dynamic scans | Static | |
| Scanner calibration | All PET/CT scanners were regularly cross-calibrated with the dose calibrator of the hospital pharmacy and the dose calibrator of the pharmacy that delivers the [^18^F]FDG. Cross-calibration was performed accord to the EARL-accreditation program and the guidelines for quality control of the Dutch Association of Nuclear Medicine (NVNG). | |
| Patient instructions | Free-breathing PET scans were acquired with the patient instructed not to move and positioned in restraining/supportive devices. | |
| Anatomical motion correction | No anatomical motion correction is performed, due to the hybrid nature of PET/CT it is assumed that the position of PET and CT with respect to the scanner’s coordinate system stays constant. | |
| Scan duration | 1.5-5 min per bed position | |
| Tube voltage ldCT | 100-140 kVp  Median: 120 kVp | |
| Tube current ldCT | 7-159 mA  Median: 50 mA | |
| Time-of-flight | 65/84 (77%) scans | |
| **Reconstruction** | | |
| In plane resolution | PET: 2.04×2.04 – 4.11×4.11 mm²  ldCT: 0.49×0.49 – 1.52×1.52 mm² | |
| Image slice thickness | PET: 2-5 mm  ldCT: 2-5 mm | |
| Image slice spacing | PET: 2-5 mm  ldCT: 2-5 mm | |
| Convolution kernel and exposure | B31f, I30f 3, I50s 3  19-99 mAs  Median: 30 mAs | |
| Reconstruction method | Line-of-response row-action maximum likelihood algorithm, BLOB-TOF algorithm, ordered-subsets expectation maximization algorithm (with point-spread-function modelling) | |
| Point spread function modelling | 30/84 (36%) scans | |
| Image corrections   - Attenuation correction - Other corrections | Attenuation correction based on low-dose CT. Correction for scatter, randoms, normalization, dead time and physical decay was applied. | |
| **Image processing – data conversion** | | |
| SUV normalisation | Body weight (in grams) | |
| Other data conversions | NA | |
| **Image processing – post acquisition processing** | | |
| Anti-aliasing | NA | |
| Noise suppression | NA | |
| Post-reconstruction smoothing filter | 5-8 mm FWHM Gaussian kernel | |
| Intensity normalisation | NA | |
| Other post-acquisition processing methods | NA | |
| **Segmentation** | | |
| Segmentation method   - Method - Number of experts, expertise, consensus strategies - Settings - Images | VOIs were delineated semi-automatically using 3DSlicer (version 4.11; www.slicer.org) and in-house built software implemented in Python 3.6.10 (Python Software Foundation, Wilmington, Delaware).  VOI were delineated on the [^18^F]FDG-PET scans using an isocontour that applies a threshold of 50% of the peak standardized uptake value (SUV_peak_), obtained using a sphere 1 cm^3^, corrected for local background [3, 4]. Boxing was applied to exclude surrounding [^18^F]FDG-avid tissues.  To apply the VOI on low-dose CT images, all VOI were resampled to the corresponding low-dose CT spacing using a nearest neighbor algorithm. No range on HU was set to exclude air and bone voxels, since high HU values might capture micro-, eggshell- and macro-calcifications, which are prevalent in both benign and malignant thyroid nodules.  VOIs were delineated by JM (Master student with 1 year of experience) and supervised by DV (nuclear medicine physician with 12 years of experience) | |
| Conversion to mask | NA | |
| **Image processing – image interpolation** | | |
| Interpolation algorithm   - Algorithm - Interpolation grid - Dimensions - Extrapolation | Images were interpolated to isotropic voxels using B-spline interpolation, with grids aligned by the input origin and only covering the VOI (PyRadiomics default). | |
| Interpolated voxel dimensions | PET: 4×4×4 mm³  ldCT: 2×2×2 mm³ | |
| **Image processing – ROI interpolation and re-segmentation** | | |
| Interpolation algorithm | NA | |
| Partially masked voxels | NA | |
| Re-segmentation methods | NA | |
| **Image processing – discretisation** | | |
| Discretisation method   - Method - Number of bins/bin size - Lowest intensity first bin | Discretisation using a fixed bin size. Bin edges were equally spaced from 0 (e.g., 0-0.5, 0.5-1, etc) and the lowest grey value was discretized into the first bin.  PET: 0.5 g/mL  ldCT: 25 HU | |
| **Image processing – image transformation** | | |
| Image filter | NA | |
| **Image biomarker computation** | | |
| Biomarker set (PyRadiomics nomenclature, if IBSI nomenclature differed, it was added in brackets) | PET and ldCT:   - First Order Statistics (18 features): 10^th^ Percentile, 90^th^ Percentile, Energy, Entropy (Intensity Histogram Entropy), Interquartile Range, Kurtosis, Maximum, Mean Absolute Deviation, Mean, Median, Minimum, Range, Robust Mean Absolute Deviation, Root Mean Squared, Skewness, Total Energy (not present in IBSI definitions), Uniformity (Intensity histogram uniformity), Variance - Shape based (14 features): Elongation, Flatness, Least Axis Length, Major Axis Length, Maximum 2D Diameter Column, Maximum 2D Diameter Row, Maximum 2D Diameter Slice, Maximum 3D Diameter, Mesh Volume (Volume), Minor Axis Length, Sphericity, Surface Area, Surface Volume Ratio, Voxel Volume (Approximate Volume) - Grey Level Cooccurrence Matrix (GLCM; 24 features): Autocorrelation, Joint Average, Cluster Prominence, Cluster Shade, Cluster Tendency, Contrast, Correlation, Difference Average, Difference Entropy, Difference Variance, Joint Energy (Angular Second Moment), Joint Entropy, Informational Measure of Correlation 1, Informational Measure of Correlation 2, Inverse Difference Moment, Inverse Difference Moment Normalized, Inverse Difference, Inverse Difference Normalized, Inverse Variance, Maximum Probability (Joint Maximum), Sum Entropy, Sum of Squares (Joint Variance), Sum Average, Maximal Correlation Coefficient - Grey Level Run Length Matrix (GLRLM; 16 features): Short Run Emphasis, Long Run Emphasis, Grey Level Non-Uniformity, Grey Level Non-Uniformity Normalized, Run Length Non-Uniformity, Run Length Non-Uniformity Normalized, Run Percentage, Grey Level Variance, Run Variance, Run Entropy, Low Grey Level Run Emphasis, High Grey Level Run Emphasis, Short Run Low Grey Level Emphasis, Short Run High Grey Level Emphasis, Long Run Low Grey Level Emphasis, Long Run High Grey Level Emphasis - Grey Level Size Zone Matrix (GLSZM; 16 features): Small Area Emphasis (Small Zone Emphasis), Large Area Emphasis (Large Zone Emphasis), Grey Level Non-Uniformity, Grey Level Non-Uniformity Normalized, Size-Zone Non-Uniformity (Zone Size Non-Uniformity), Size-Zone Non-Uniformity Normalized (Zone Size Non-Uniformity Normalized), Zone Percentage, Grey Level Variance, Zone Variance (Zone Size Variance), Zone Entropy (Zone Size Entropy), Low Grey Level Zone Emphasis, High Grey Level Zone Emphasis, Small Area Low Grey Level Emphasis (Small Zone Low Grey Level Emphasis), Small Area High Grey Level Emphasis (Small Zone High Grey Level Emphasis), Large Area Low Grey Area Emphasis (Large Zone Low Grey Level Emphasis), Large Area High Grey Level Emphasis (Large Zone High Grey Level Emphasis) - Grey Level Dependence Matrix (GLDM; 14 features): Small Dependence Emphasis (Low Dependence Emphasis), Large Dependence Emphasis (High Dependence Emphasis), Grey Level Non-Uniformity, Dependence Non-Uniformity (Dependence Count Non-Uniformity), Dependence Non-Uniformity Normalized (Dependence Count Non-Uniformity Normalized), Grey Level Variance, Dependence Variance (Dependence Count Variance), Dependence Entropy (Dependence Count Entropy), Low Grey Level Emphasis (Low Grey Level Count Emphasis), High Grey Level Emphasis (High Grey Level Count Emphasis), Small Dependence Low Grey Level Emphasis (Low Small Dependence Low Grey Level Emphasis), Small Dependence High Grey Level Emphasis (Low Dependence High Grey Level Emphasis), Large Dependence Low Grey Level Emphasis (High Dependence Low Grey Level Emphasis), Large Dependence High Grey Level Emphasis (High Dependence Low Grey Level Emphasis) - Neighbouring Grey Tone Difference Matrix (NGTDM; 5 features): Coarseness, Contrast, Busyness, Complexity, Strength   PET:   - Total Lesion Glycolysis | |
| IBSI compliance | Yes | |
| Robustness | Not assessed | |
| Software availability | PyRadiomics 2.1.2 in Python 3.6.10 (Python Software Foundation, Wilmington, Delaware) | |
| **Image biomarker computation – texture parameters** | | |
| Texture matrix aggregation | GLCM and GLRLM: 3D: average; GLSZM, GLDM and NGTDM: 3D | |
| Distance weighting | No weighting | |
| Cooccurrence matrix symmetry | Symmetric | |
| Cooccurrence matrix distance | Chebyshev distance of 1 | |
| Size zone matrix linkage distance | Chebyshev distance of 1 | |
| Distance zone matrix linkage distance | NA | |
| Distance zone matrix distance norm | NA | |
| Neighbouring grey tone difference matrix distance | Chebyshev distance of 1 | |
| Grey level dependence matrix distance | Chebyshev distance of 1 | |
| Grey level dependence matrix coarseness | 0 | |
| **Machine learning and radiomic analysis** | | |
| Diagnostic and prognostic modelling | Documented in the next section using the Transparent Reporting of a multivariable prediction model for Individual Prognosis or Diagnosis (TRIPOD, version October 1 2020) Checklist: Prediction Model Development and Validation [5]. | |
| Comparison with known factors | NA | |
| Multicollinearity | Unsupervised dimension reduction using redundancy filtering (Spearman correlation matrix threshold = 0.95) and factor analysis was performed in the folds. Features were scaled (centred around 0, variance of 1) to avoid that features with the largest scale would dominate the analysis. Redundancy filtering was performed by creating a correlation matrix of all features. For each row, the number of times a features exhibits an absolute correlation above the threshold (≥0.95) is collected in a vector. The feature with the most absolute correlations exceeding the threshold is represented by most other features and is thus removed. A new correlation matrix is generated with the remaining features and the process is iteratively repeated until the generated correlation matrix does not contain absolute correlations above the threshold. Factor analysis, a statistical method to project the feature space on a lower-dimensional latent meta-feature (i.e., factor) space, was executed on the redundancy filtered correlation matrix using an orthogonal rotation. In this way, the first factor explained the largest possible variance in the dataset; succeeding factors explained the largest variance in orthogonal directions. The sampling adequacy of the model was determined by the Kaiser-Meier-Olkin (KMO) measure, which was required to be between 0.9 and 1.0. The definitions of the factors in the model were determined based on the underlying clusters of features in the different folds. One factor was selected for every ten subjects in the training set. Dimensionality reduction was performed in FMradio (Factor Modeling for Radiomics Data, version 1.1.1) in R (version 3.6.0) [6]. | |
| Model availability | The models generated during and/or analysed during the current study are available from the corresponding author on reasonable request. | |
| Data availability | The datasets generated during and/or analysed during the current study are available from the corresponding author on reasonable request. | |
| **TRIPOD checklist** | | |
| Objectives | Evaluate whether [^18^F]FDG-PET/CT radiomics could improve the preoperative differentiation of cytologically indeterminate thyroid nodules. | |
| Source of data | All patients who participated in a randomized controlled multicentre trial (ClinicalTrials.gov NCT02208544, and the Netherlands Trial Register NL50166.091.14) on the efficacy of [^18^F]FDG-PET/CT in cytologically indeterminate thyroid nodules, were evaluated (start accrual: 1 July 2015, end accrual 16 October 2018, end follow-up: 21 December 2019). | |
| Participants | Participants: 80 patients with [^18^F]FDG PET-positive lesions and PET/CT scans following the EANM-guidelines were included in the radiomic analysis. Patient characteristics are described in table 1 of the manuscript. | |
| Outcome | Accurate preoperative differentiation between benign and malignant/borderline nodules, with the ultimate aim to prevent futile diagnostic surgery for benign nodules. | |
| Predictors | Radiomic features as extracted from the [^18^F]FDG PET and low-dose CT scans. Radiomic features are specified above. | |
| Statistical analysis | An elastic net regression classifier was trained and evaluated in a 20-times repeated random split (80% training set, 20% test set). In each split, unsupervised dimensionality reduction of the radiomic feature set in the training set is performed using redundancy filtering (Spearman correlation matrix threshold = 0.9) and factor. One factor was selected for every ten subjects in the training set. Factors for the training and test set were calculated and the factors of the training set were used as input for the elastic net regression classifier. The predictive performance of the model is expressed as the mean AUC of the ROC curve over the 20 splits for the test sets. The 95% confidence intervals (CI) were constructed using a corrected resampled t-test. Classification models were trained on PET features only and on PET and CT features combined and subgroup analyses for nodules with Hürthle cell and non-Hürthle cell cytology were performed. | |
| Model performance, test AUC (95% CI) | [^18^F]FDG-positive nodules (N=84) | PET/CT: 0.461 (0.289-0.633)  PET: 0.445 (0.290-0.600) |
|  | Non-Hürthle cell (N=28) | PET/CT: 0.466 (0.236-0.695)  PET: 0.519 (0.298-0.740) |
|  | Hürthle cell (N=56) | PET/CT: 0.537 (0.208-0.867)  PET: 0.694 (0.461-0.926) |
|  | Additional details of the model can be found in Table 7 of the manuscript. | |

**Supplementary table 3:**

**Baseline characteristics of included as compared to excluded patients**

|  | **Included patients (n=123)** | **Excluded patients (n=9)** |  |
| --- | --- | --- | --- |
|  | n (%) | n (%) | *p* |
| Female sex | 102 (82.9%) | 5 (56%) | 0.07^a^ |
| Age (years) (mean ± SD) | 55.0 ± 13.4 | 46.8 ± 16.1 | 0.09^b^ |
| *Ultrasound characteristics* |  |  |  |
| Solitary nodule | 87 (71%) | 6 (67%) | 0.72^a^ |
| Dominant nodule in multinodular disease | 36 (29%) | 3 (33%) |  |
| Size (mm) (median, IQR) | 35 (22-44) | 35 (23-39) | 0.77^c^ |
| Suspicious characteristics* | 49 (40%) | 6 (67%) | 0.16^a^ |
| Solid hypoechoic nodule | 34 (28%) | 4 (44%) | 0.28^a^ |
| Taller-than-wide shape | 1 (1%) | 0 (0%) | 1^a^ |
| Irregular margins | 9 (7%) | 0 (0%) | 1^a^ |
| Microcalcifications | 14 (11%) | 2 (22%) | 0.3^a^ |
| *Cytology* |  |  |  |
| Bethesda III | 55 (45%) | 5 (56%) | 0.73^a^ |
| Bethesda IV | 68 (55%) | 4 (44%) |  |
| FN/SFN | 39 (32%) | 2 (22%) | 1^a^ |
| HCN/SHCN | 29 (24%) | 2 (22%) |  |
| *Thyroid function* |  |  |  |
| TSH, mU/L (median, IQR)** | 1.70 (1.08-2.40) | 1.40 (0.95-2.00) | 0.36^c^ |
| fT4, pmol/L (median, IQR)*** | 14.6 (13.2-16.6) | 14.6 (13.3-16.1) | 0.69^c^ |
|  |  |  |  |
| Surgery | 100 (81.3%) | 9 (100%) | 0.36^a^ |
| *Malignant histopathology* | 24 (20%) | 1 (11%) | 1^a^ |
| PTC | 5 | 1 (11%) |  |
| FVPTC | 4 | 0 (0%) |  |
| FTC, minimally invasive | 6 | 0 (0%) |  |
| HCC, minimally invasive | 5 | 0 (0%) |  |
| DTC not otherwise specified | 1 | 0 (0%) |  |
| PDTC | 1 | 0 (0%) |  |
| MTC | 2 | 0 (0%) |  |
| *Borderline histopathology* | 9 (7%) | 0 (0%) | 1^a^ |
| NIFTP | 5 | 0 (0%) |  |
| FT-UMP, Hürthle cell type | 3 | 0 (0%) |  |
| Paraganglioma | 1 | 0 (0%) |  |
| *Benign histopathology* | 67 (54%) | 8 (89%) | 0.08^a^ |
| Follicular adenoma | 28 | 3 (33%) |  |
| Hürthle cell adenoma | 13 | 1 (11%) |  |
| Hyperplastic nodule | 26 | 4 (44%) |  |
| No surgery, unsuspicious on ultrasound f/u | 23 (19%) | 0 (0%) |  |
| [^18^F]FDG-positive | 84 (68%) | 7 (78%) | 0.72^a^ |

AUS/FLUS, atypia of undetermined significance or follicular lesions of undetermined significance. DTC, differentiated thyroid carcinoma. FN/SFN, (suspicious for a) follicular neoplasm. fT4, free thyroxine. FTC, follicular thyroid carcinoma. FT-UMP, follicular tumour of uncertain malignant potential. FVPTC, follicular variant PTC. HCC, Hürthle cell carcinoma. HCN/SHCN, (suspicious for a) Hürthle cell neoplasm. IQR, interquartile range. MTC, medullary thyroid carcinoma. PDTC, poorly differentiated thyroid carcinoma. PTC, papillary thyroid carcinoma. NIFTP, non-invasive follicular thyroid neoplasm with papillary-like nuclear features. SD, standard deviation. TSH, thyroid stimulating hormone.

^a^: Fisher’s exact test. ^b^: independent samples t-test. ^c^: Mann-Whitney U test.

**Supplementary table 4:**

**Baseline characteristics of included patients (3 groups)**

|  | **Non-Hürthle cell (n=94)** | | **Hürthle cell** |  |
| --- | --- | --- | --- | --- |
|  | **AUS/FLUS (n=55)** | **FN/SFN (n=39)** | **HCN/SHCN (n=29)** |  |
|  | n (%) | n (%) | n (%) | *p^d^* |
| Female sex | 50 (91%) | 29 (74%) | 23 (79%) | 0.09^e^ |
| Age (years) (mean ± SD) | 54.3 ± 13.6 | 55.4 ± 12.2 | 55.7 ± 14.8 | 0.88^f^ |
| *Ultrasound characteristics* |  |  |  |  |
| Solitary nodule | 38 (69%) | 26 (67%) | 23 (79%) | 0.49^e^ |
| Dominant nodule in multinodular disease | 17 (31%) | 13 (33%) | 6 (21%) |  |
| Size (mm) (median, IQR) | 35 (25-46) | 35 (19-41) | 33 (23-43) | 0.75^g^ |
| Suspicious characteristics^a^ | 19 (35%) | 18 (46%) | 12 (41%) | 0.52^e^ |
| Solid hypoechoic nodule | 14 (25%) | 13 (33%) | 7 (24%) | 0.63^e^ |
| Taller-than-wide shape | 0 (0%) | 0 (0%) | 1 (3%) | 0.20^e^ |
| Irregular margins | 7 (13%) | 1 (3%) | 1 (3%) | 0.12^e^ |
| Microcalcifications | 3 (5%) | 7 (18%) | 4 (14%) | 0.15^e^ |
| *Thyroid function* |  |  |  |  |
| TSH, mU/L (median, IQR)^b^ | 1.70 (1.10-2.40) | 1.62 (0.90-2.30) | 1.70 (1.35-3.00) | 0.47^g^ |
| fT4, pmol/L (median, IQR)^c^ | 14.8 (13.1-16.7) | 14.4 (13.1-16.7) | 14.2 (13.3-15.6) | 0.85^g^ |
| Diagnostic surgery | 43 (78%) | 31 (79%) | 26 (90%) | 0.41^e^ |
| *Malignant histopathology* | 6 (11%) | 12 (31%) | 6 (21%) | 0.06^e^ |
| PTC | 2 | 3 | 0 |  |
| FVPTC | 2 | 2 | 0 |  |
| FTC, minimally invasive | 2 | 4 | 0 |  |
| HCC, minimally invasive | 0 | 0 | 5 |  |
| DTC not otherwise specified | 0 | 0 | 1 |  |
| PDTC | 0 | 1 | 0 |  |
| MTC | 0 | 2 | 0 |  |
| *Borderline histopathology* | 4 (7%) | 2 (5%) | 3 (10%) | 0.72^e^ |
| NIFTP | 3 | 1 | 1 |  |
| FT-UMP, Hürthle cell type | 1 | 0 | 2 |  |
| Paraganglioma | 0 | 1 | 0 |  |
| *Benign histopathology* | 33 (60%) | 17 (44%) | 17 (59%) | 0.25^e^ |
| Follicular adenoma | 14 | 13 | 1 |  |
| Hürthle cell adenoma | 4 | 0 | 9 |  |
| Hyperplastic nodule | 15 | 4 | 7 |  |
| No surgery, unsuspicious on ultrasound f/u | 12 (22%) | 8 (21%) | 3 (10%) | 0.41^d^ |
| [^18^F]FDG-positive | 31 (56%) | 25 (64%) | 28 (97%) | **<0.001^d^** |
| SUV_max_ nodule (g/mL) (median, IQR) | 3.4 (2.3-6.9) | 3.4 (2.3-4.9) | 12.3 (5.8-33.2) | **<0.001^g^** |
| SUV_peak_ nodule (g/mL) (median, IQR) | 2.9 (2.0-5.3) | 2.5 (2.0-4.2) | 12.3 (5.8-33.2) | **<0.001^g^** |
| SUV_max_ thyroid background (g/mL) (median, IQR) | 1.9 (1.7-2.5) | 2.0 (1.8-2.4) | 1.8 (1.6-2.2) | 0.28^g^ |
| SUV_max_-ratio (median, IQR) | 1.6 (1.2-3.2) | 1.6 (1.1-2.6) | 3.9 (6.4-12.1) | **<0.001^g^** |
| SUV_peak_-ratio (median, IQR) | 1.3 (0.9-2.3) | 1.2 (0.9-2.2) | 4.7 (2.8-10.7) | **<0.001^g^** |

AUS/FLUS, atypia of undetermined significance or follicular lesions of undetermined significance. DTC, differentiated thyroid carcinoma. FN/SFN, (suspicious for a) follicular neoplasm. fT4, free thyroxine. FTC, follicular thyroid carcinoma. FT-UMP, follicular tumour of uncertain malignant potential. FVPTC, follicular variant PTC. HCC, Hürthle cell carcinoma. HCN/SHCN, (suspicious for a) Hürthle cell neoplasm. IQR, interquartile range. MTC, medullary thyroid carcinoma. PDTC, poorly differentiated thyroid carcinoma. PTC, papillary thyroid carcinoma. NIFTP, non-invasive follicular thyroid neoplasm with papillary-like nuclear features. SD, standard deviation. TSH, thyroid stimulating hormone. ^a^: Suspicious ultrasound characteristics were defined as presence of at least one of the following characteristics: marked hypoechogenicity (in a solid nodule), irregular shape (i.e., taller-than-wide), irregular margins, and/or presence of microcalcifications.

^b^: The reference range for TSH is 0.4–4.0 mU/L.

^c^: The reference range for fT4 is approximately 10–25 pmol/L (sex and age dependent).

^d^: the three subgroups are compared to each other; p values of the indicated statistical tests are reported. ^e^: Pearson’s chi-squared test. ^f^: one-way ANOVA. ^g^: Kruskal-Wallis test, indicating statistically significant differences between AUS/FLUS and HCN/SHCN groups and between FN/SFN and HCN/SHCN groups for the SUV_max_, SUV_peak_, SUV_max_-ratio, and SUV_peak_-ratio (p<0.001 for all analyses) but not between AUS/FLUS and FN/SFN groups (p=0.94, p=0.88, p=0.86, and p=0.82, respectively).

**Supplementary table 5: Baseline characteristics of malignant/borderline nodules as compared to benign nodules**

|  | **Malignant/borderline (n=33)** | **Benign (n=90)** |  |
| --- | --- | --- | --- |
|  | n (%) | n (%) | *p* |
| Female sex | 21 (64%) | 81 (90%) | **<0.001^d^** |
| Age (years) (mean ± SD) | 56.2 ± 14.7 | 54.5 ± 13.0 | 0.57^e^ |
| *Ultrasound characteristics* |  |  |  |
| Solitary nodule | 22 (67%) | 65 (72%) | 0.55^d^ |
| Dominant nodule in multinodular disease | 11 (33%) | 25 (28%) |  |
| Size (mm) (median, IQR) | 36 (25-46) | 34 (22-42) | 0.32^f^ |
| Suspicious characteristics^a^ | 17 (52%) | 32 (36%) | 0.11^d^ |
| Solid hypoechoic nodule | 12 (36%) | 22 (24%) | 0.19^d^ |
| Taller-than-wide shape | 0 (0%) | 1 (1%) | 1^g^ |
| Irregular margins | 1 (3%) | 8 (9%) | 0.44^d^ |
| Microcalcifications | 6 (18%) | 8 (9%) | 0.20^d^ |
| *Cytology* |  |  |  |
| Bethesda III | 10 (30%) | 45 (50%) | 0.052^d^ |
| Bethesda IV | 23 (70%) | 45 (50%) |  |
| FN/SFN | 14 (61%) | 25 (56%) | 0.68^d^ |
| HCN/SHCN | 9 (39%) | 20 (44%) |  |
| *Thyroid function* |  |  |  |
| TSH, mU/L (median, IQR)^b^ | 1.78 (1.40-2.40) | 1.60 (0.94-2.43) | 0.27^f^ |
| fT4, pmol/L (median, IQR)^c^ | 13.7 (12.6-15.7) | 14.8 (13.4-16.9) | **0.04^f^** |
| Diagnostic surgery | 33 (100%) | 67 (74%) | **0.001^d^** |
| *Malignant histopathology* | 24 (20%) |  |  |
| PTC | 5 |  |  |
| FVPTC | 4 |  |  |
| FTC, minimally invasive | 6 |  |  |
| HCC, minimally invasive | 5 |  |  |
| DTC not otherwise specified | 1 |  |  |
| PDTC | 1 |  |  |
| MTC | 2 |  |  |
| *Borderline histopathology* | 9 (7%) |  |  |
| NIFTP | 5 |  |  |
| FT-UMP, Hürthle cell type | 3 |  |  |
| Paraganglioma | 1 |  |  |
| *Benign histopathology* |  | 67 (54%) |  |
| Follicular adenoma |  | 28 |  |
| Hürthle cell adenoma |  | 13 |  |
| Hyperplastic nodule |  | 26 |  |
| No surgery, unsuspicious on ultrasound f/u |  | 23 (19%) |  |
| [^18^F]FDG-positive | 31 (94%) | 53 (59%) | **<0.001^d^** |

DTC, differentiated thyroid carcinoma. FN/SFN, (suspicious for a) follicular neoplasm. fT4, free thyroxine. FTC, follicular thyroid carcinoma. FT-UMP, follicular tumour of uncertain malignant potential. FVPTC, follicular variant PTC. HCC, Hürthle cell carcinoma. HCN/SHCN, (suspicious for a) Hürthle cell neoplasm. IQR, interquartile range. MTC, medullary thyroid carcinoma. PDTC, poorly differentiated thyroid carcinoma. PTC, papillary thyroid carcinoma. NIFTP, non-invasive follicular thyroid neoplasm with papillary-like nuclear features. SD, standard deviation. TSH, thyroid stimulating hormone.

^a^: Suspicious ultrasound characteristics were defined as presence of at least one of the following characteristics: marked hypoechogenicity (in a solid nodule), irregular shape (i.e., taller-than-wide), irregular margins, and/or presence of microcalcifications. ^b^: The reference range for TSH is 0.4–4.0 mU/L. ^c^: The reference range for fT4 is approximately 10–25 pmol/L (sex and age dependent). ^d^: Pearson’s chi-squared test. ^e^: independent samples t-test. ^f^: Mann-Whitney U test. ^g^: Fisher’s exact test.

**Supplementary table 6: Differences in SUV metrices between malignant/borderline and benign nodules in AUS/FLUS and FN/SFN nodules**

|  | **Malignant/ borderline** | **Benign** | p |
| --- | --- | --- | --- |
| **AUS/FLUS (n=55)** | **n=10** | **n=45** |  |
| SUV_max_ nodule, g/mL | 5.7 (3.1-10.4) | 3.3 (2.2-6.8) | 0.10 |
| SUV_peak_ nodule, g/mL | 4.0 (2.4-8.5) | 2.9 (1.8-4.8) | 0.16 |
| SUV_max_ thyroid background, g/mL | 1.8 (1.7-2.1) | 2.0 (1.8-2.6) | 0.24 |
| SUV_max_-ratio | 2.2 (1.6-6.4) | 1.5 (1.1-3.0) | **0.03** |
| SUV_peak_-ratio | 1.5 (1.3-5.3) | 1.3 (0.9-2.3) | 0.06 |
| **FN/SFN (n=39)** | **n=9** | **n=20** |  |
| SUV_max_ nodule, g/mL | 7.9 (3.4-16.1) | 2.9 (2.2-4.0) | **0.001** |
| SUV_peak_ nodule, g/mL | 7.1 (2.4-12.1) | 2.3 (1.9-3.1) | **0.003** |
| SUV_max_ thyroid background, g/mL | 2.0 (1.6-2.5) | 2.0 (1.9-2.4) | 0.55 |
| SUV_max_-ratio | 2.5 (1.6-8.8) | 1.2 (1.0-1.8) | **<0.001** |
| SUV_peak_-ratio | 2.1 (1.2-6.9) | 1.0 (0.9-1.5) | **0.004** |

SUV values are presented as median (interquartile range, IQR) and compared between groups using the Mann-Whitney U test. AUS/FLUS, atypia of undetermined significance or follicular lesions of undetermined significance. CI, confidence interval. FN/SFN, (suspicious for a) follicular neoplasm. HCN/SHCN, (suspicious for a) Hürthle cell neoplasm. SUV, standardized uptake value.

**Supplementary table 7: Threshold analysis and diagnostic accuracy for AUS/FLUS and FN/SFN nodules**

|  | **SUV**  **cut-off** | **TP** | **FP** | **TN** | **FN** | **Sensitivity,**  **% (95% CI)** | **Specificity,**  **% (95% CI)** | **NPV,**  **% (95% CI)** | **PPV,**  **% (95% CI)** | **benign call rate,**  **% (95% CI)** |
| --- | --- | --- | --- | --- | --- | --- | --- | --- | --- | --- |
| **Visual assessment** |  |  |  |  |  |  |  |  |  |  |
| AUS/FLUS (n=55) |  | 9 | 22 | 23 | 1 | 90.0 (55.5-99.7) | 51.1 (35.8-66.3) | 95.8 (78.9-99.9) | 29.0 (14.2-48.0) | 43.6 (30.3-57.7) |
| FN/SFN (n=39) |  | 13 | 12 | 13 | 1 | 92.9 (66.1-99.8) | 52.0 (31.3-72.2) | 92.9 (66.1-99.8) | 52.0 (31.3-72.2) | 35.9 (21.2-52.8) |
| **Quantitative analysis** |  | | | | | | | | | |
| **AUS/FLUS (n=55)** |  |  |  |  |  |  |  |  |  |  |
| SUV_max_ nodule, g/mL | 2.1 | 10 | 34 | 11 | 0 | 100 (69.2-100) | 24.4 (12.9-39.5) | 100 (71.5-100) | 22.7 (11.5-37.8) | 24.4 (12.9-39.5) |
| SUV_peak_ nodule, g/mL | 1.5 | 10 | 39 | 6 | 0 | 100 (69.2-100) | 13.3 (5.1-26.8) | 100 (54.1-100) | 20.4 (10.2-34.3) | 13.3 (5.1-26.8) |
| SUV_max_-ratio | 1.2 | 10 | 26 | 19 | 0 | 100 (69.2-100) | 42.2 (27.7-57.8) | 100 (82.4-100) | 27.8 (14.2-45.2) | 42.2 (27.7-57.8) |
| SUV_peak_-ratio | 0.9 | 10 | 32 | 13 | 0 | 100 (69.2-100) | 28.9 (16.4-44.3) | 100 (75.3-100) | 23.8 (12.1-39.5) | 28.9 (16.4-44.3) |
| **FN/SFN (n=39)** |  |  |  |  |  |  |  |  |  |  |
| SUV_max_ nodule, g/mL | 2.4 | 13 | 16 | 9 | 1 | 92.9 (66.1-99.8) | 36.0 (18.0-57.5) | 90.0 (55.5-99.7) | 44.8 (26.4-64.3) | 25.6 (13.0-42.1) |
| SUV_peak_ nodule, g/mL | 2.0 | 13 | 18 | 7 | 1 | 92.9 (66.1-99.8) | 28.0 (12.1-49.4) | 87.5 (47.3-99.7) | 41.9 (24.5-60.9) | 20.5 (9.3-36.5) |
| SUV_max_-ratio | 1.3 | 13 | 12 | 13 | 1 | 92.9 (66.1-99.8) | 52.0 (31.3-72.2) | 92.9 (66.1-99.8) | 52.0 (31.3-72.2) | 35.9 (21.2-52.8) |
| SUV_peak_-ratio | 0.9 | 13 | 20 | 5 | 1 | 92.9 (66.1-99.8) | 20.0 (6.8-40.7) | 83.3 (35.9-99.6) | 39.4 (22.9-57.9) | 15.4 (5.9-30.5) |

AUS/FLUS, atypia of undetermined significance or follicular lesions of undetermined significance. CI, confidence interval. FN, false negative. FN/SFN, (suspicious for a) follicular neoplasm. FP, false positive. NPV, negative predictive value. PPV, positive predictive value. SUV, standardized uptake value. TN, true negative. TP, true positive.

**Supplementary figure 1: ROC curves of quantitative [^18^F]FDG-PET/CT analysis in AUS/FLUS and FN/SFN nodules**

1. ****
2. ****

ROC curves for SUV_max_ (blue line), SUV_peak_ (green), SUV_max_-ratio (purple), and SUV_peak_-ratio (red) in (**a)** AUS/FLUS (n=55), (**b**) FN/SFN (n=39) nodules. **a:** In AUS/FLUS nodules, the AUCs for the SUV_max_, SUV_peak_, SUV_max_-ratio, and SUV_peak_-ratio were 0.667 (95% CI, 0.490-0.843), 0.642 (0.448-0.837), 0.722 (0.566-0.879), and 0.691 (0.519-0.863), respectively. **b:** In FN/SFN nodules, these AUCs were 0.803 (95% CI, 0.651-0.955), 0.780 (0.614-0.946), 0.814 (0.672-0.956), and 0.777 (0.607-0.947), respectively.

**Supplementary table 8: Results of ROC curve analysis in [^18^F]FDG-positive non-Hürthle cell nodules (n=56)**

|  | **n** | **Malignant/borderline** | **Benign** | **p-value** |
| --- | --- | --- | --- | --- |
| **[^18^F]FDG-positive non-Hürthle cell nodules** | **56** | **n=22** | **n=34** |  |
| SUV_max_ nodule, g/mL |  | 6.4 (3.4-15.8) | 4.7 (3.8-7.7) | 0.25 |
| SUV_peak_ nodule, g/mL |  | 5.4 (2.7-11.4) | 4.0 (2.9-5.6) | 0.25 |
| SUV_max_ thyroid background, g/mL |  | 1.9 (1.7-2.4) | 2.0 (1.8-2.5) | 0.29 |
| SUV_max_-ratio |  | 2.5 (1.7-8.5) | 2.5 (1.7-3.2) | 0.21 |
| SUV_peak_-ratio |  | 2.1 (1.3-5.7) | 2.0 (1.3-2.4) | 0.25 |

CI, confidence interval. SUV, standardized uptake value.

SUV values are presented as median (IQR) and compared between groups using the Mann-Whitney U test.

**Supplementary table 9: Threshold analysis and diagnostic accuracy in [^18^F]FDG-positive non-Hürthle cell nodules (n=56)**

|  | **AUC (95% CI)** | **SUV cut-off** | **TP** | **FP** | **TN** | **FN** | **Sensitivity,**  **% (95% CI)** | **Specificity,**  **% (95% CI)** | **NPV,**  **% (95% CI)** | **PPV,**  **% (95% CI)** | **benign call rate,**  **% (95% CI)** |
| --- | --- | --- | --- | --- | --- | --- | --- | --- | --- | --- | --- |
| SUV_max_ nodule, g/mL | 0.592 (0.425-0.759) | 2.5 | 21 | 33 | 1 | 1 | 95.5 (77.2-99.9) | 2.9 (0.1-15.3) | 50.0 (1.3-98.7) | 38.9 (25.9-53.1) | 3.6 (0.4-12.3) |
| SUV_peak_ nodule, g/mL | 0.591 (0.420-0.762) | 2.0 | 21 | 33 | 1 | 1 | 95.5 (77.2-99.9) | 2.9 (0.1-15.3) | 50.0 (1.3-98.7) | 38.9 (25.9-53.1) | 3.6 (0.4-12.3) |
| SUV_max_-ratio | 0.600 (0.436-0.765) | 1.3 | 21 | 32 | 2 | 1 | 95.5 (77.2-99.9) | 5.9 (0.7-19.7) | 66.7 (9.4-99.2) | 39.6 (26.5-54.0) | 5.4 (1.1-14.9) |
| SUV_peak_-ratio | 0.591 (0.424-0.758) | 1.0 | 21 | 33 | 1 | 1 | 95.5 (77.2-99.9) | 2.9 (0.1-15.3) | 50.0 (1.3-98.7) | 38.9 (25.9-53.1) | 3.6 (0.4-12.3) |

AUC, area under the receiver operating characteristic curve. CI, confidence interval. FN, false negative. FP, false positive. NPV, negative predictive value. PPV, positive predictive value. SUV, standardized uptake value. TN, true negative. TP, true positive.

**Supplementary table 10:**

**Radiomic analysis: dimensionality reduction and predictive performance of radiomic models for all [^18^F]FDG-positive nodules, non-Hürthle cell nodules and Hürthle cell nodules.**

|  | **All [^18^F]FDG-positive nodules (n=84)** | | **Non-Hürthle cell nodules (n=56)** | | **Hürthle cell nodules (n=28)** | | **[^18^F]FDG-positive nodules > 64 voxels (n=66)** |
| --- | --- | --- | --- | --- | --- | --- | --- |
|  | **PET/CT** | **PET** | **PET/CT** | **PET** | **PET/CT** | **PET** | **PET** |
| **KMO, median (range)^a^** | 0.967 (0.965-0.972) | 0.938 (0.927-0.946) | 0.977 (0.975-0.980) | 0.964 (0.960-0.970) | 0.978 (0.973-0.981) | 0.952 (0.943-0.961) | 0.946 (0.934-0.954) |
| **Cumulative variance all factors, median (range)^a^** | 0.654 (0.637-0.680) | 0.796 (0.774-0.821) | 0.518 (0.482-0.560) | 0.673 (0.618-0.712) | 0.306 (0.273-0.342) | 0.515 (0.466-0.563) | 0.691 (0.677-0.720) |
| **Retained factors^b^** | 6 factors:  - Nodule size  - Variance of the intensity histogram on CT  - High intensity on PET  - Fine textures on CT  - Mutual information between different regions on CT  - Large areas on PET | 6 factors:  - Entropy of the intensity histogram  - Nodule size  - High intensity on PET  - Variance in area size  - Total lesion glycolysis  - Small areas with low grey levels | 4 factors:  - High intensity on PET  - Nodule size  - Variance of the intensity histogram on CT  - Variance in voxel dependency on CT | 4 factors:  - High intensity on PET  - Entropy of the intensity histogram  - Nodule size  - Large areas | 2 factors:  - Variance of the intensity histogram on CT  - Entropy of the intensity histogram on PET | 2 factors:  - Nodule size  Entropy of the intensity histogram | 5 factors:  - High intensity on PET  - Nodule size  - Entropy of the intensity histogram  - Variance in area size  - Total lesion glycolysis |
| **Test AUC (95% CI)** | 0.461 (0.289-0.633) | 0.445 (0.290-0.600) | 0.466 (0.236-0.695) | 0.519 (0.298-0.740) | 0.537 (0.208-0.867) | 0.694 (0.461-0.926) | 0.421 (0.303-0.538) |

KMO, Kaiser-Meier Olkin measure, AUC, area under the receiver operating characteristic curve.

^a^: The KMO and the cumulative variance of all factors are expressed as median and range of all 20 folds.

^b^: One factor was retained per ten subjects in the training set.

**Supplementary figure 2: ROC curves of the radiomic analysis, PET/CT model**

**
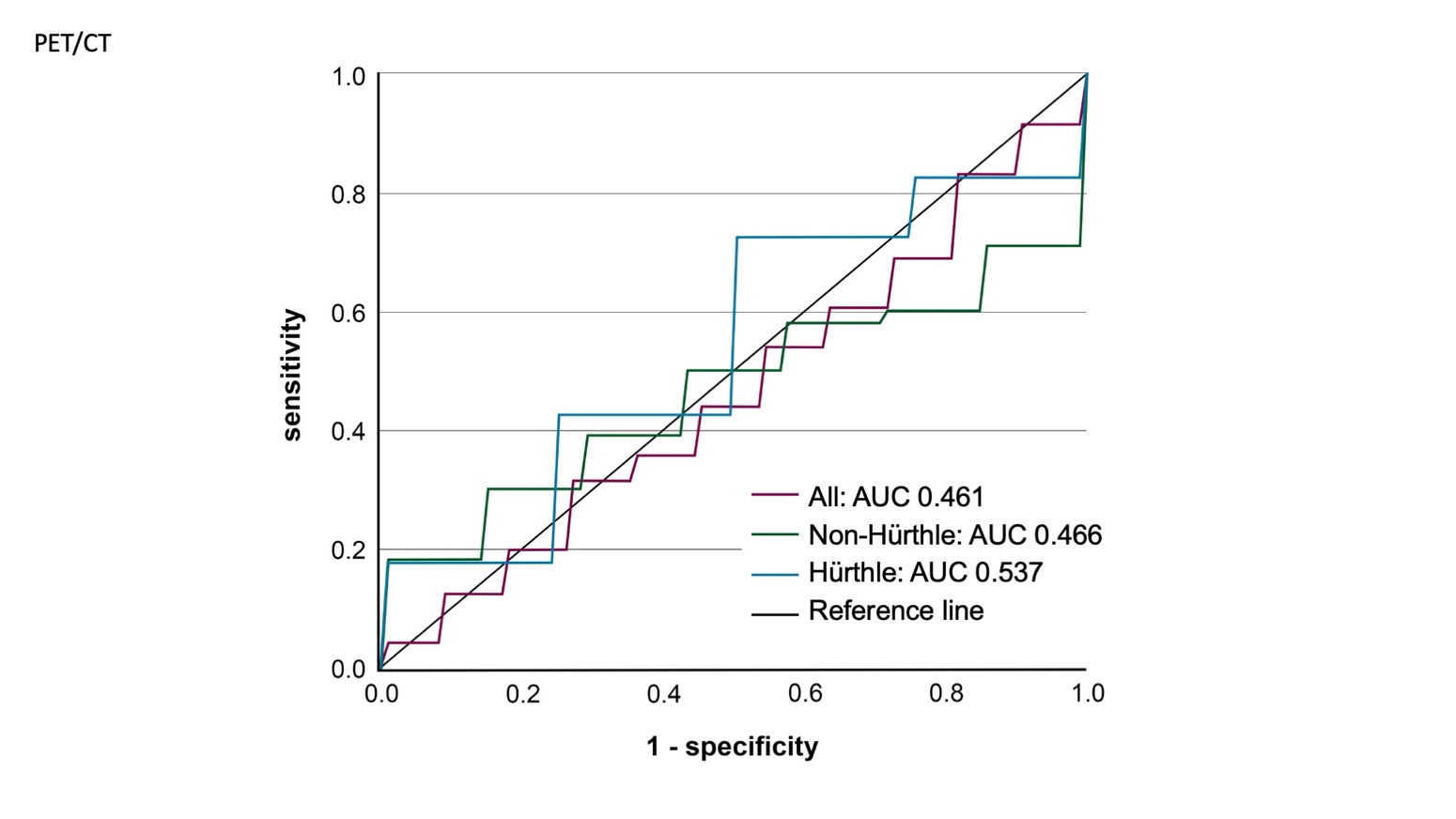
**

ROC curves for the PET/CT model of the radiomic analysis in all nodules (purple line), non-Hürthle cell nodules (green), and Hürthle cell nodules (blue). 95% CI of the AUC are presented in Supplementary table 10.

**References**

1. Zwanenburg A, Leger S, Vallières M, Löck S. Image biomarker standardisation initiative. arXiv 2019;1612.07003.

2. Boellaard R, Delgado-Bolton R, Oyen WJ, Giammarile F, Tatsch K, Eschner W, et al. FDG PET/CT: EANM procedure guidelines for tumour imaging: version 2.0. Eur J Nucl Med Mol Imaging. 2015;42:328–54. https://doi.org/10.1007/s00259-014-2961-x.

3. Frings V, van Velden FH, Velasquez LM, Hayes W, van de Ven PM, Hoekstra OS, et al. Repeatability of metabolically active tumor volume measurements with FDG PET/CT in advanced gastrointestinal malignancies: a multicenter study. Radiology. 2014;273:539-48. https://doi.org/10.1148/radiol.14132807.

4. Wahl RL, Jacene H, Kasamon Y, Lodge MA. From RECIST to PERCIST: Evolving Considerations for PET response criteria in solid tumors. Journal of nuclear medicine : official publication, Society of Nuclear Medicine. 2009;50 Suppl 1:122S-50S. https://doi.org/10.2967/jnumed.108.057307.

5. Moons KG, Altman DG, Reitsma JB, Ioannidis JP, Macaskill P, Steyerberg EW, et al. Transparent Reporting of a multivariable prediction model for Individual Prognosis or Diagnosis (TRIPOD): explanation and elaboration. Annals of internal medicine. 2015;162:W1-73. https://doi.org/10.7326/M14-0698.

6. Peeters CFW, Übelhör C, Mes SW, Martens RM, Koopman T, Graaf Pd, et al. Stable prediction with radiomics data. ArXiv. 2019;abs/1903.11696.
